# Four New Antibacterial Xanthones from the Marine-Derived Actinomycetes *Streptomyces caelestis*

**DOI:** 10.3390/md10112571

**Published:** 2012-11-20

**Authors:** Ling-Li Liu, Ying Xu, Zhuang Han, Yong-Xin Li, Liang Lu, Pok-Yui Lai, Jia-Liang Zhong, Xian-Rong Guo, Xi-Xiang Zhang, Pei-Yuan Qian

**Affiliations:** 1 Division of Life Science, The Hong Kong University of Science and Technology, Clear Water Bay, Kowloon, Hong Kong SAR, China; Email: leonie@ust.hk (L.-L.L.); boxuying@ust.hk (Y.X.); zhuanghan@ust.hk (Z.H.); liyongxing@ust.hk (Y.-X.L.); lluaa@ust.hk (L.L.); cycylai@gmail.com (P.-Y.L.); 2 Shanghai Institute of Pharmaceutical Industry, Shanghai 200040, China; Email: jialiang.zhong@yahoo.com.cn; 3 Advanced Nano-Fabrication, Imaging and Characterization Core Lab, King Abdullah University of Science and Technology, Thuwal 23955-6900, Saudi Arabia; Email: xianrong.guo@kaust.edu.sa (X.-R.G.); xixiang.zhang@kaust.edu.sa (X.-X.Z.)

**Keywords:** *Streptomyces caelestis*, citreamicin, antibacterial, MRSA

## Abstract

Four new polycyclic antibiotics, citreamicin *θ* A (**1**), citreamicin *θ* B (**2**), citreaglycon A (**3**), and dehydrocitreaglycon A (**4**), were isolated from marine-derived *Streptomyces caelestis*. The structures of these compounds were elucidated by 1D and 2D NMR spectra. All four compounds displayed antibacterial activity against *Staphylococcus haemolyticus*, *Staphylococcus aureus*, and *Bacillus subtillis*. Citreamicin *θ* A (**1**), citreamicin *θ* B (**2**) and citreaglycon A (**3**) also exhibited low MIC values of 0.25, 0.25, and 8.0 μg/mL, respectively, against methicillin-resistant *Staphylococcus aureus* (MRSA) ATCC 43300.

## 1. Introduction

The marine environment is a plentiful source of bioactive natural products with potential for development as new pharmaceutical agents. To date, the success rate of drug discovery from the marine world is seven approved drugs out of 22,000 discovered molecular entities, which is approximately 1.7 to 3.3-fold higher than the industry average [1]. Among the diverse spectrum of marine organisms, culturable marine bacteria are prime candidates due to their production of bioactive natural products for possible use in chemical and clinical research [2].

Methicillin-Resistant *Staphylococcus aureus* (MRSA), a traditional nosocomial opportunistic pathogen, has spread from hospitals to the community in the last few decades [3]. Compared with methicillin-susceptible *S. aureus *bacteremia, MRSA bacteremia cases are associated with greater lengths of hospital stay, significantly higher mortality, and increased costs, posing a heavy burden on healthcare systems [4,5,6]. In the United States, the standardized mortality rate from MRSA infection in 2005 was 6.3 per 100,000 [7]. MRSA contains a mutant gene that manufactures a protein that protects the bacterium from the binding of penicillin [8]. Due to the current prevalence of multidrug-resistant bacteria such as MRSA in many countries, the discovery of new antibiotics with novel modes of action is crucial to tackle the threat of multidrug-resistant bacteria [9]. 

Citreamicins are polycyclic xanthones isolated from *Streptomyces*, and have been reported to show antibacterial activity against a variety of Gram-positive bacteria, including MRSA and vancomycin-resistant *Enterococcus faecalis* (VRE). To date, 10 citreamicins have been reported by several research groups [10,11,12,13,14]. Citreamicins share a common 5/6/6/6/6/6/6 seven-ring system, and identifying their respective structures is usually difficult due to the existence of multiple quaternary carbon atoms.

With the aim of discovering novel antibacterial natural products from marine bacteria, we examined a rarely explored area of rich biodiversity—the Red Sea. We performed a systematic biological and chemical analysis of the secondary metabolites produced by bacteria isolated from the Red Sea based on various bioassays, ultra performance liquid chromatography-mass spectrometry (UPLC-MS) analysis, and UV profile analysis. One of the hits resulting from our efforts was identified by its 16S rRNA gene as the strain *Streptomyces caelestis*. The genus *Streptomyces* belongs to the actinomycete family, which is responsible for producing about half of all of the discovered secondary metabolites known to have broad biological activity, such as antibacterial, anticancer, and anti-inflammatory activity [15,16,17,18,19]. Bioassay-Guided fractionation of the *S. caelestis* extract led to the isolation of four new compounds (**1**–**4**), all of which showed antibacterial activity against *Staphylococcus haemolyticus*, *Staphylococcus aureus*, and *Bacillus subtillis*. Citreamicin *θ* A (**1**), citreamicin *θ* B (**2**), and citreaglycon A (**3**) also exhibited low minimum inhibitory concentration (MIC) values of 0.25, 0.25, and 8.0 μg/mL, respectively, against methicillin-resistant strain *Staphylococcus aureus* (MRSA) ATCC 43300. Compounds **1** and **2** also displayed cytotoxic activity against HeLa cells. Here, we report the fermentation, isolation, structural elucidation, and bioactivity evaluation of these isolated compounds from *S. caelestis*.

## 2. Results and Discussion

**Figure 1:**
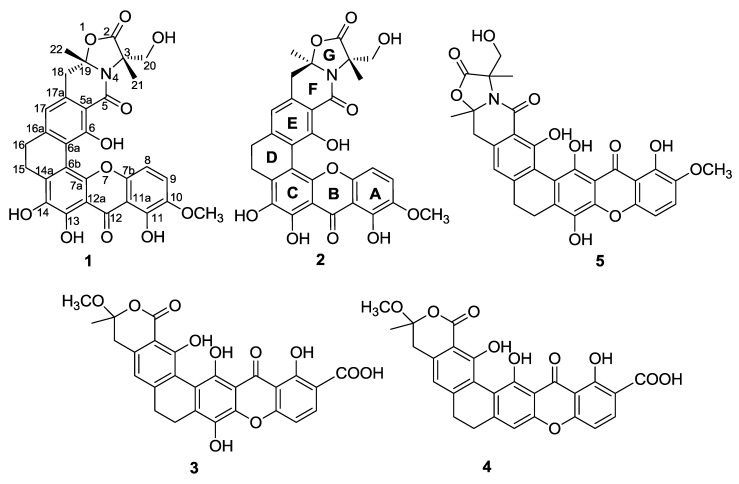
Structures of compounds **1**–**5**.

**Figure 2:**
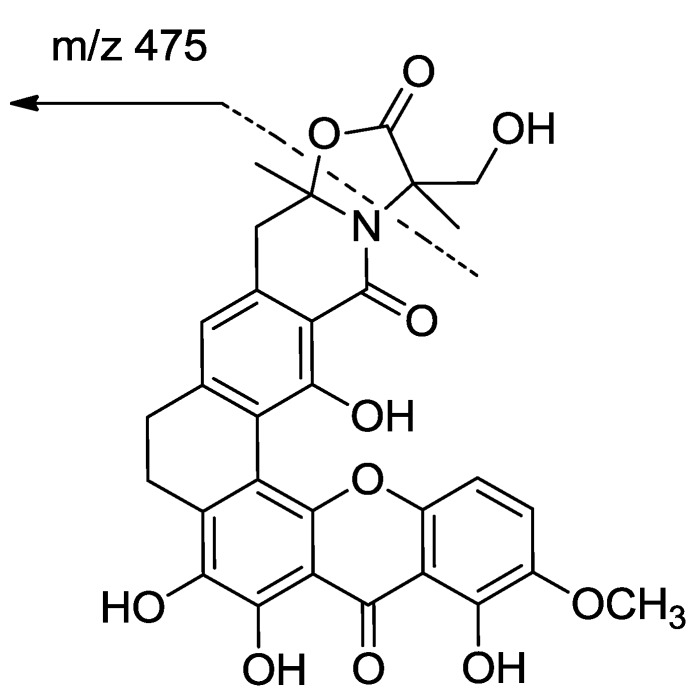
Ion source collision-induced dissociation electrospray ionization mass spectrometry fragmentations of compounds **1** and **2**.

Compound **1** was isolated as a red crystal with the molecular formula C_30_H_25_NO_11_, based on a high resolution electrospray ionization mass spectroscopy (HRESIMS) of 576.1508 for the [M + H]^+^ value (calcd 576.1506), indicating 19 degrees of unsaturation. The UV spectrum showed the existence of conjugation based on absorption at λ_max_ 237, 278, 323, and 441 nm. Analysis of the ^13^C NMR spectra indicated the presence of three carbonyls (*δ*_C_ 183.1, 173.7, 166.3), including a ketone at *δ*_C_ 183.1; 18 sp^2^ carbons in the down field region (three methines (*δ*_C_ 122.6, 118.2, 107.7) and fifteen quaternary carbons); and two sp^3^ quaternary carbons (*δ*_C_ 93.6, 65.5), four methylene groups (*δ*_C_ 61.6, 40.4, 29.4, 23.6), two methyl groups (*δ*_C_ 26.5, 19.2) and one methoxy (*δ*_C_ 57.3) in the high field region. Together, these signals reveal a skeleton of xanthones very similar to that identified in citreamicin *ε* (**5**) [13] (Figure 1). A fragment ion at *m*/*z* 475 in ion source collision-induced dissociation electrospray ionization mass spectrometry (ISCID ESIMS) corresponding to citeamicin *ε* (**5**) without the G ring ion was also observed (Figure 2). 

Further comparison of the 1D and 2D NMR data of compound **1** and citreamicin *ε *[13] indicated that they are structural isomers with the same molecular weight and similar chemical shifts. Heteronuclear multiple bond correlation (HMBC) correlations from the methyl group at *δ*_H_ 1.61 (H-21) to carbons at *δ*_C_ 173.7 (C-2), 65.5 (C-3), and 61.6 (C-20), together with HMBC correlations from *δ*_H_ 1.67 (H-22) to *δ*_C_ 40.4 (C-18) and 93.6 (C-19), reveal the G ring. However, as rings B and C are constituted by quaternary carbons, it was difficult to obtain an unambiguous structure through 2D NMR analysis. To determine the exact difference in the constituent parts of compound **1** and citreamicin *ε*, we attempted to culture a single crystal of compound **1**. From the crystal data [20], we were able to identify two ortho-phenolic hydroxyls at positions 13 and 14 and to determine the planar structure of compound **1** as that shown in Figure 1. 

Compound **2** was also isolated as a red crystal with an identical UV profile to that of compound **1 **with absorption at λ_max_ 237, 278, 323, and 441 nm. Its molecular formula of C_30_H_25_NO_11_ ([M + H]^+^ 576.1500 calcd 576.1506) obtained from HRESIMS and the ^1^H and ^13^C NMR data (Table 1) are very similar to those of **1**, with the significant differences being the methylene signals at position 18 (*δ*_H_ 3.37 in **1** and *δ*_H_ 3.45 in **2**) and at position 20 (*δ*_H_ 3.55/4.31, *δ*_C_ 61.6 in **1** and *δ*_H_ 3.69/4.10, *δ*_C_ 63.4 in **2**) in the 1D NMR spectra. Further analysis of the 2D NMR spectra of compound **2** showed that a proton signal at *δ*_H_ 7.54 (H-9) had a H^1^-H^1^ correlation (H^1^-H^1^ COSY) to *δ*_H_ 6.82 (H-8) and HMBC correlations to *δ*_C_ 145.5 (C-7), 149.1 (C-10), 143.3 (C-11). Together, these data elucidate the construction of the A and B rings. HMBC correlations from the methyl group at *δ*_H_ 1.61 (H-21) to carbons at *δ*_C_ 173.4 (C-2), 65.6 (C-3) and 63.4 (C-20), together with HMBC correlations from *δ*_H_ 1.69 (H-22) to *δ*_C_ 40.0 (C-18) and 93.7 (C-19) reveal the G ring. The similar HMBC correlations in **2** to those observed in **1** suggest that they are a pair of diastereomers.

**Table 1:** ^1^H and ^13^C NMR data for compounds **1** and **2** in DMSO-*d*_6_.

Position	1		2	
	*δ*_C_^a^	*δ*_H_ (*J* in Hz) ^b^	*δ*_C_^a^	*δ*_H_ (*J* in Hz) ^b^
2	173.7, qC		173.4, qC	
3	65.5, qC		65.6, qC	
5	166.3, qC		166.0, qC	
5a	109.6, qC		109.2, qC	
6	147.2, qC		146.9, qC	
6a	118.3, qC		118.1, qC	
6b	102.6, qC		102.1, qC	
7a	150.6, qC		150.2, qC	
7b	145.9, qC		145.5, qC	
8	107.7, CH	6.87, d (8.8)	107.2, CH	6.82, d (8.9)
9	122.6, CH	7.57, d (8.8)	122.3, CH	7.54, d (8.9)
10	149.5, qC		149.1, qC	
11	143.7, qC		143.3, qC	
11a	106.4, qC		105.9, qC	
12	183.1, qC		182.7, qC	
12a	108.3, qC		107.9, qC	
13	159.2, qC		158.7, qC	
14	137.3, qC		136.7, qC	
14a	134.6, qC		134.9, qC	
15	23.6, CH_2_	4.09, m	23.2, CH_2_	4.05, m
16	29.4, CH_2_	3.72, m	29.6, CH_2_	3.69, m
16a	142.3, qC		142.0, qC	
17	118.2, CH	6.90, s	117.8, CH	6.87, s
17a	141.8, qC		141.4, qC	
18	40.4, CH_2_	3.37, m	40.0, CH_2_	3.45, m
19	93.6, qC		93.7, qC	
20	61.6, CH_2_	3.55, d (10.9)	63.4, CH_2_	3.69, d (11.1)
		4.31, d (10.9)		4.10, d (11.1)
21	19.2, CH_3_	1.61, s	18.6, CH_3_	1.61, s
22	26.5, CH_3_	1.67, s	25.0, CH_3_	1.69, s
-OCH_3_	57.3, CH_3_	3.85, s	56.9, CH_3_	3.82, s
6-OH		12.01, s		12.01, s
11-OH		11.77, s		11.75, s
14-OH		9.23, s		9.23, s

^a^ Recorded at 500 MHz; ^b^ Recorded at 125 MHz.

**Figure 3:**
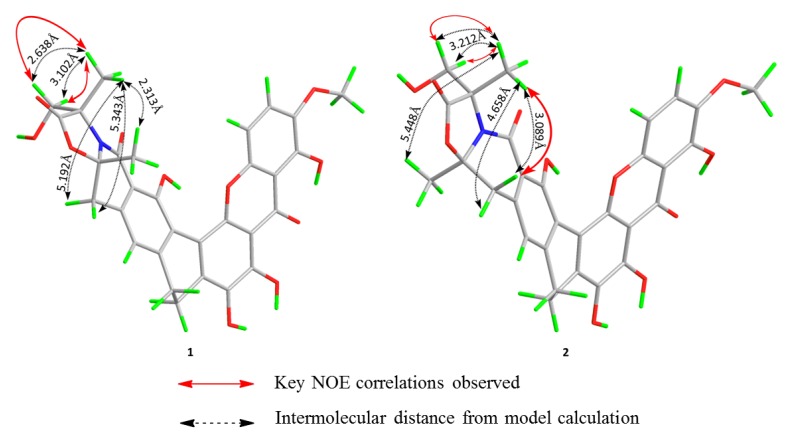
Key nuclear overhauser effect correlations of compounds **1** and **2 **and the optimized 3D model structures.

As both compounds **1** and **2 **possess two stereogenic centers, there were two possibilities regarding the directions of the two methyl groups at C-3 and C-19. The relative configuration of **2** was determined from the nuclear overhauser effect spectroscopy (NOESY). The structures of **1** and **2** were optimized by the molecular modeling (MM2) force field and the calculated distance between the H_3_-21 (*δ*_H_ 1.61) and H-18 (*δ*_H_ 3.45) are shown in Figure 3. The distances between protons beyond 4 Å would give no or only very weak nuclear overhauser effect (NOE) signals [21]. Although, the two protons at position 18 were not distinguished, the distance between H_3_-21 and one of the H-18 is 4.658 Å that is beyond the cutoff of 4 Å [21]. The NOE correlation between H_3_-21 (*δ*_H_ 1.61) and H-18 (*δ*_H_ 3.45) revealed that they are on the same side of the molecule, which indicates that the methyl groups at positions C-3 and C-19 have opposite orientations (Figure 3).

**Figure 4:**
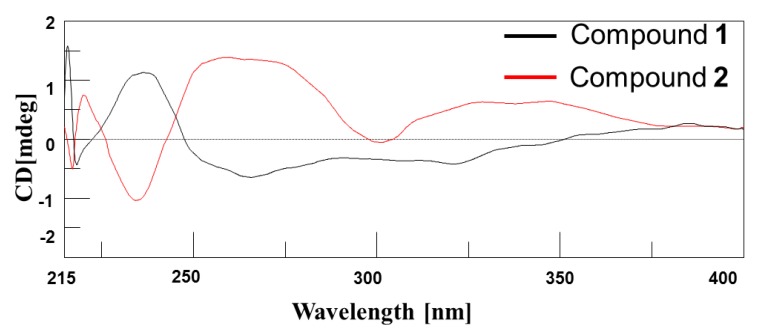
The circular dichroism spectra of compounds **1** and **2**.

The circular dichroism (CD) spectrum of compound **1** displayed a negative Cotton effect with weak minima at short wavelengths and a maximum at higher wavelengths [λ_max_ 220 nm (Δ*ε *−0.41), λ_max_ 248 nm (Δ*ε *+1.14)] (Figure 4). However, the CD spectrum of compound **2** showed an almost opposite Cotton effect to that of compound **1**. The CD spectra of compounds **1** and **2** confirmed that these two compounds were diastereomers [22,23].

Compound **3** has the molecular formula of C_27_H_20_O_11_, as established by the HRESIMS of the pseudomolecular ion peak at *m/z* 521.1079 [M + H]^+^ (calcd for 521.1084). The UV spectrum displayed absorption at λ_max_ 238, 298, 412 nm, which is similar to the findings for compound **1**. The ^1^H NMR spectrum revealed the presence of four phenolic protons (*δ*_H_ 9.29, 10.3, 11.7, 12.6), three aromatic protons (*δ*_H_ 6.82, 7.49, 7.63), one methoxy group (*δ*_H_ 3.31), one methyl (*δ*_H_ 1.66), and three methylene groups (Table 2). The ^13^C NMR spectrum displayed 27 carbon signals, which were classified as three carbonyls (*δ*_C_ 167.5, 169.0, 180.9); nineteen carbons between *δ*_C_ 160 and 100, indicating multiple aromatic rings; three methylene (*δ*_C_ 23.1, 29.6, 36.8), one methoxy (*δ*_C_ 50.1); and one methyl group (*δ*_C_ 21.7). Compound **3** had the same xanthone skeleton as compound **1**, but without the G ring.

**Table 2:** ^1^H and ^13^C NMR data for compounds **3** and **4** in DMSO-*d_6_*.

Position	3		4	
	*δ*_C_^a^	*δ*_H_ (*J* in Hz) ^b^	*δ*_C_^a^	*δ*_H_ (*J* in Hz) ^b^
2	169.0, qC		169.2, qC	
2a	106.6, qC		106.7, qC	
3	158.4, qC		158.0, qC	
3a	118.3, qC		118.8, qC	
3b	112.5, qC		110.5, qC	
4	150.2, qC		149.9, qC	
4a	106.6, qC		106.7, qC	
5	180.9, qC		181.2, qC	
5a	117.4, qC		117.9, qC	
6	150.1, qC		149.9, qC	
7	116.7, qC		115.9, qC	
8	125.1, CH	7.49, d (9.1)	125.6, CH	7.45, d (9.1)
9	119.3, CH	7.63, d (9.1)	119.3, CH	7.37, d (9.1)
9a	148.6, qC		147.1, qC	
10a	144.1, qC		144.9, qC	
11	137.5, qC		118.7, qC	6.90, s
11a	132.3, qC		135.9, qC	
12	23.1, CH_2_	2.24, m	23.0, CH_2_	2.23, m
		3.31, overlap		3.25, m
13	29.6, CH_2_	2.63, m	29.2, CH_2_	2.64, m
13a	148.8, qC		148.9, qC	
14	117.5, CH	6.82, s	117.9, CH	6.84, s
14a	137.4, qC		137.7, qC	
15	36.8, CH_2_	3.16, m	37.8, CH_2_	3.22, m
		3.38, m		3.41, m
16	106.1, qC		106.4, qC	
17	21.7, CH_3_H	1.66, s	22.4, CH_3_	1.69, s
18	167.5, qC		167.6, qC	
–OCH_3_	50.1	3.31, s	50.1	3.35, s
3-OH		11.7, s		11.8, s
4-OH		12.6, s		12.8, s
11-OH		9.29, s		9.22, s
18-OH		10.3, s		10.3, s

^a^ Recorded at 500 MHz; ^b^ Recorded at 125 MHz.

**Figure 5:**
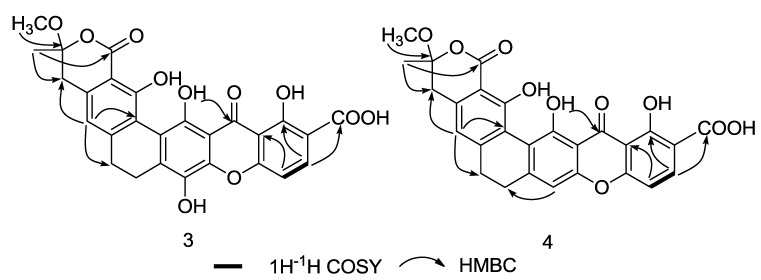
Key ^1^H-^1^H COSY and HMBC correlations of compounds **3** and **4**.

The HMBC spectrum of **3** revealed correlations from *δ*_H_ 3.31 and *δ*_H_ 1.66 to *δ*_C_ 106.1 (C-16), indicating the attachment of the methyl and methoxy group at C-16. The downfield chemical shift of C-16 suggests that this quaternary carbon attaches to an additional oxygen. The construction of the F ring was verified by an HMBC correlation from *δ*_H_ 1.66 to carbonyl carbon *δ*_C_ 169.0 (C-2) and *δ*_C_ 36.8 (C-15). HMBC correlation from *δ*_H_ 7.49 (H-8) to *δ*_C_ 167.5 (C-18) provided evidence for the presence of a carboxylic acid group at C-7 (Figure 5), and the planar structure of compound **3** was thus determined to be that shown in Figure 1. However, the relative structure of **3** at C-16 remains unconfirmed due to the limited signals in the NOESY spectrum. Furthermore, since the single crystal of compound **3** was not obtainable, and no CD signal was observed, the absolute configuration of compound **3** is still undetermined.

Compound **4** has the molecular formula C_27_H_20_O_10_, as deduced from its HRESIMS data ([M + H]^+^ at *m/z* 505.1130, calcd for 505.1135), which is 16 Dalton less than the value for compound **3**. Comparison of the ^1^H and ^13^C NMR spectral data (Table 2) indicates close similarities between **4** and **3**, with the only difference occurring at C-11. The phenolic hydroxyl group at C-11 in compound **3** is replaced by an aromatic proton in compound **4**, as confirmed by an HMBC correlation in compound **4** from *δ*_H_ 6.90 (H-11) to *δ*_C_ 22.4 (C-12). These findings revealed the structure of **4** to be that shown in Figure 1. The relative configuration at C-16 was again undetermined due to insufficient NOESY key correlations. The absolute configuration of **4** was still unconfirmed due to the absent of single crystal data and the CD signal.

The antibacterial and cytotoxic activity of compounds **1**–**4** was evaluated. The diastereomers—compounds **1** and **2**—showed similar antibacterial activity against four strains: *B. subtillis* 769, *S. haemolyticus* UST950701-004, *S. aureus* UST950701-005, and *S. aureus *ATCC 43300, with MIC values of ≤1.0 μg/mL. Compounds **3** and **4** also displayed similar biological activity against these bacterial strains, except that compound **4** revealed no activity against *S. aureus *ATCC 43300. The cytotoxic activity of these four compounds was also assessed against HeLa cells by the standard 3-(4,5-dimethylthiazol-2-yl)-2,5-diphenyltetrazolium bromide (MTT) assay [24]. Compounds **1** and **2** displayed significant cytotoxic activity at nanomolar concentrations, whereas compounds **3 **and **4** showed no activity (Table 3). Compounds **1** and **2** displayed much stronger antibacterial activity than compounds **3** and **4**, suggesting that the five-member nitrogen heterocycle, which is absent from compounds **3** and **4**, is essential for activity. The five-member nitrogen heterocycle is quite similar to that in oxazolidinones, which are an approved class of antibiotics [25,26]. The mode of action of compounds **1** and **2** with the five-member nitrogen heterocycle is currently being studied.

**Table 3:** Antibacterial and cytotoxic activity of compounds **1**–**4**.

Compound	Antibacterial (MIC, μg/mL)				Cytotoxicity (IC_50_μg/mL)
	*Staphylococcus haemolyticus* UST950701-004	*Staphylococcus aureus* UST950701-005	*Bacillus subtillis* 769	*Staphylococcus aureus *ATCC43300	HeLa cells
1	0.5	1.0	0.25	0.25	0.055
2	0.5	1.0	0.25	0.25	0.072
3	8.0	16.0	8.0	8.0	>40
4	8.0	16.0	8.0	NA	>40
Penicillin G	0.13	0.25	0.13	NA	NT
Streptomycin	8.0	8.0	8.0	NA	NT
Cisplatin	NT	NT	NT	NT	18.14

NA means MIC > 50 μg/mL, NT means NOT test.

## 3. Experimental Section

### 3.1. General Experimental Procedures

The ^1^H, ^13^C, and 2D NMR spectral data were obtained with Varian Inova 500 MHz and Bruker 700 MHz NMR spectrometers. The ESI and high-resolution mass spectra were acquired from ultra-performance liquid chromatography-time of fly-mass spectrometry (UPLC-TOF-MS). The UPLC system was a Waters ACQUITY UPLC system (Waters, Manchester, UK) coupled with a Bruker microTOF-q II mass spectrometer (Bruker Daltonics GmbH, Bremen, Germany). The X-ray diffraction study was carried out using a Bruker SMART APEX-2 CCD (Bruker, Rheinstetten, Germany), and the semi-preparative HPLC was performed using a C18 (2) column that was 5 μm and 250 × 10 mm in size. The optical density (OD) measurements in the experiments were recorded at 595 nm on a Thermo scientific Multiskan FC multiplate photometer (Waltham, MA, USA).

### 3.2. Sample Collection and Microbial Material

The *Streptomyces *sp. was isolated from the coastal water of the Red Sea by the side of a fish market near Jeddah (21′29.622N 39′09.617E). The total genomic DNA preparation of the strain was carried out following the procedure in the literature [27]. The Blast program was used to assess the DNA similarities in the NCBI Databases. The strain displayed 99% similarity with *Streptomyces caelestis* strain NRRL 2418. The complete 16S rDNA sequence data suggests that the strain is most closely related to *Streptomyces caelestis* [28]. The sequence of the strain has been deposited in GenBank with the accession number: JX204833.

### 3.3. Fermentation, Extraction, and Isolation

The strain was cultured in 37 × 1.0 L volume of media (10 g/L of starch, 2 g/L of peptone, 4 g/L of yeast extract and 20 g/L sea salt) at 23 °C for 5 days. Each flask contained about 100 glass beads (3 mm in diameter). The spent fermentation culture (37.0 L) was filtered with 8 layers of cheesecloth. The broth was then extracted with ethyl acetate, and the mycelia were extracted with acetone and methanol (1:2 v/v). The extracts from the broth and the mycelia were combined and partitioned between water and hexane three times. The resulting aqueous residue was further extracted with ethyl acetate (EtOAc). The separation of the EtOAc soluble constituents (15.3 mg) was achieved by reverse phase C18 flash chromatography, and elution with solvent mixtures of H_2_O–MeOH (9:1), H_2_O–MeOH (7:3), H_2_O–MeOH (5:5), H_2_O–MeOH (3:7), H_2_O–MeOH (9:1), and 100% MeOH. The fraction that was eluted with H_2_O–MeOH (3:7, v/v) was evaporated, to yield a residue (labeled Fraction (Fr.) B, 630 mg). The Fr. B fraction was then subjected to Sephadex LH-20 eluted with MeOH to yield 14 sub-fractions (named Fr. B-1 to Fr. B-14). The final purification of Fr. B-6 was achieved by reverse-phase semi-preparative HPLC and elution with MeCN–H_2_O (50:50, (v/v), flow rate: 3 mL/min), to give compounds **1** (2.3 mg) and **2** (3.1 mg) at a retention time of 24.0 min and 29.4 min, respectively. The Fr. B-13 was further purified by the same column and eluted with MeCN–H_2_O (37:63–50:50, (v/v)) to obtain pure compounds **3** (10.2 mg) and **4 **(7.4 mg).

Citreamicin *θ* A (**1**): red crystal; [α]^25^_D_ +59 (*c* 0.02, MeOH); mp 341.1–349.1 °C; UV λ_max_(MeOH) nm 237, 278, 323, 441; ^1^H and ^13^C NMR data, see Table 1: HRESIMS *m*/*z* 576.1508 [M + H]^+^ (calcs for C_30_H_25_NO_11_ 576.1506).

Citreamicin *θ* B (**2**): red crystal; [α]^25^_D_ −43 (*c* 0.02, MeOH); mp 341.1–349.1 °C; UV λ_max_ (MeOH) nm 237, 278, 323, 441; ^1^H and ^13^C NMR data, see Table 1: HRESIMS *m*/*z* 576.1500 [M + H]^+^ (calcs for C_30_H_25_NO_11_ 576.1506).

Citreaglycon A (**3**): red powder; UV λ_max_ (MeOH) nm 238, 298, 412; ^1^H and ^13^C NMR data, see Table 2: HRESIMS *m*/*z* 521.1079 [M + H]^+^ (calcs for C_27_H_20_O_11_ 521.1084).

Dehydrocitreaglycon A (**4**): red powder; UV λ_max_ (MeOH) nm 237, 298, 411; ^1^H and ^13^C NMR data, see Table 2: HRESIMS *m*/*z* 505.1130 [M + H]^+^ (calcs for C_27_H_20_O_10_ 505.1135).

### 3.4. Antibacterial Assays

The minimum inhibitory concentration values (MIC) of compounds **1**–**4** were determined using a modification of the microdilution method described [29]. In brief, the strains *B. subtillis* 769, *S. haemolyticus* UST950701-004, *S. aureus* UST950701-005, and *S. aureus *ATCC 43300 were inoculated in Lysogeny broth (LB) (10 g of tryptone, 5 g of yeast extract, 10 g of NaCl, 1 L of dd H_2_O) and were incubated at 28 °C for 12 h. Stock solution of the samples were prepared at 40 mg/mL in DMSO and then further diluted with LB broth to varying concentrations in 96-well plates. The bacteria were incubated at 28 °C overnight. Cell growth was checked by measuring the optical density at 595 nm, with Penicillin G and Streptomycin as positive controls.

### 3.5. Cytotoxic Assays

The cytotoxicity tests were carried out according to the method previously described by Li *et al.* [30]. Briefly, HeLa cells were seeded and cultured in 96-well plates 12 h before the addition of the test samples. The compounds were dissolved in DMSO and diluted in the assay media. After incubation of the test samples for 48 h, the cell viability was assayed by the MTT method.

## 4. Conclusions

Four new xanthones were isolated from a marine-derived *Streptomyces caelestis*. Compounds **1** and **2**, named citreamicin *θ* A and B, are a pair of diasteromers that possess a 5/6/6/6/6/6/6 seven-ring system. It is the first report of the citreamicins with such a unique ring system that is bending with an acute angle. The similar MIC values of citreamicin *θ* A (**1**) and B (**2**) to those of *B. subtillis*, *S. haemolyticus*, and *S. aureus*, and the MRSA strain indicate that these two diasteromers possess equal antibacterial activity. Citreaglycon A and dehydrocitreaglycon A showed weaker antibacterial activity than citreamicin *θ* A (**1**) and citreamicin *θ* B (**2**). The greater antibacterial activity of citreamicin *θ* A (**1**) and B (**2**) may be due to the five-member nitrogen heterocycle. The role of this nitrogen heterocycle in citreamicins merits further study.

## Supplementary Files

Supplementary File 1: Supplementary Information (PDF, 1628 KB)

Supplementary File 2: CIF-Document (CIF, 24 KB)
