# Supplementary material for: Four New Antibacterial Xanthones from the Marine-Derived Actinomycetes Streptomyces caelestis
Source: Mar Drugs. 2012 Nov 20;10(11):2571–83. doi: 10.3390/md10112571 (PMC3509536; doi:10.3390/md10112571)

Supplementary Information

Figure S1. Key HMBC correlations of compounds **1** and **2** 2
Figure S2. ^{1}H NMR spectrum of **1** in DMSO-d_6 2
Figure S3. ^{13}C NMR spectrum of **1** in DMSO-d_6 3
Figure S4. HSQC spectrum of **1** in DMSO-d_6 3
Figure S5. HMBC spectrum of **1** in DMSO-d_6 4
Figure S6. NOESY spectrum of **1** in DMSO-d_6 4
Figure S7. HRESIMS spectrum of **1** 5
Figure S8. ^{1}H NMR spectrum of **2** in DMSO-d_6 6
Figure S9. ^{13}C NMR spectra of **2** in DMSO-d_6 6
Figure S10. HSQC spectrum of **2** in DMSO-d_6 7
Figure S11. HMBC spectrum of **2** in DMSO-d_6 7
Figure S12. NOESY spectrum of **2** in DMSO-d_6 8
Figure S13. HRESIMS spectrum of **2** 9
Figure S14. 3D mode and the key NOE correlations of compounds **1** and **2** 10
Figure S15. ^{1}H NMR spectrum of **3** in DMSO-d_6 11
Figure S16. ^{13}C NMR spectrum of **3** in DMSO-d_6 11
Figure S17. HSQC spectrum of **3** in DMSO-d_6 12
Figure S18. HMBC spectrum of **3** in DMSO-d_6 12
Figure S19. HRESIMS spectrum of **3** 13
Figure S20. ^{1}H NMR spectrum of **4** in DMSO-d_6 14
Figure S21. ^{13}C NMR spectrum of **4** in DMSO-d_6 14
Figure S22. HSQC spectrum of **4** in DMSO-d_6 15
Figure S23. HRESIMS spectrum of **4** 15
Figure S24. HRESIMS spectrum of **4** 16

Figure S1. Key HMBC correlations of compounds **1** and **2**.

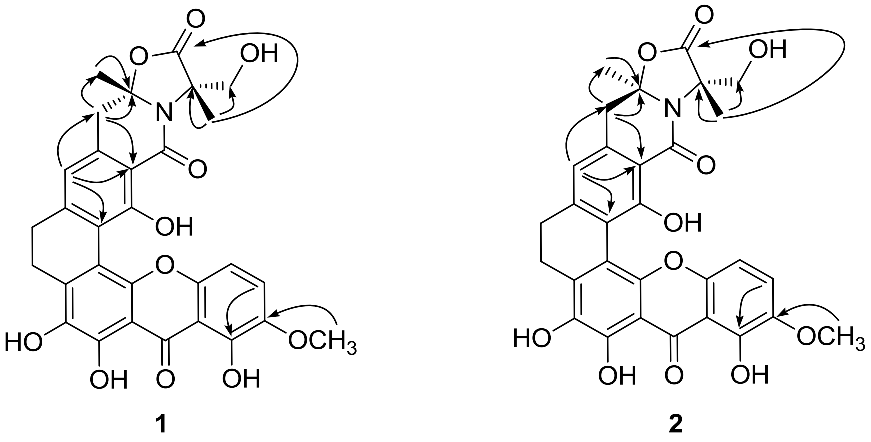

Figure S2. ^{1}H NMR spectrum of **1** in DMSO-d_6.

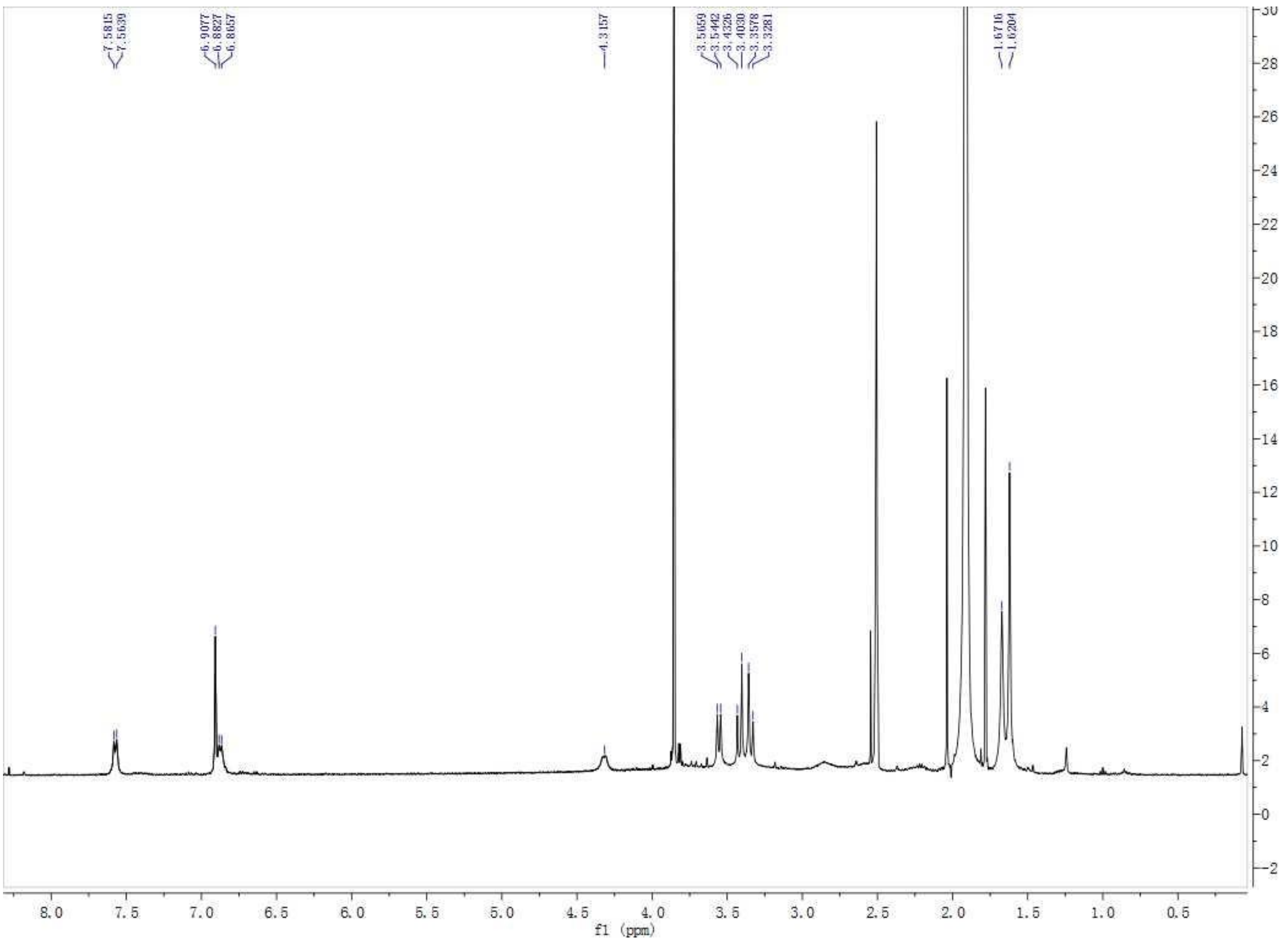

Figure S3. ^{13}C NMR spectrum of **1** in DMSO-d_6.

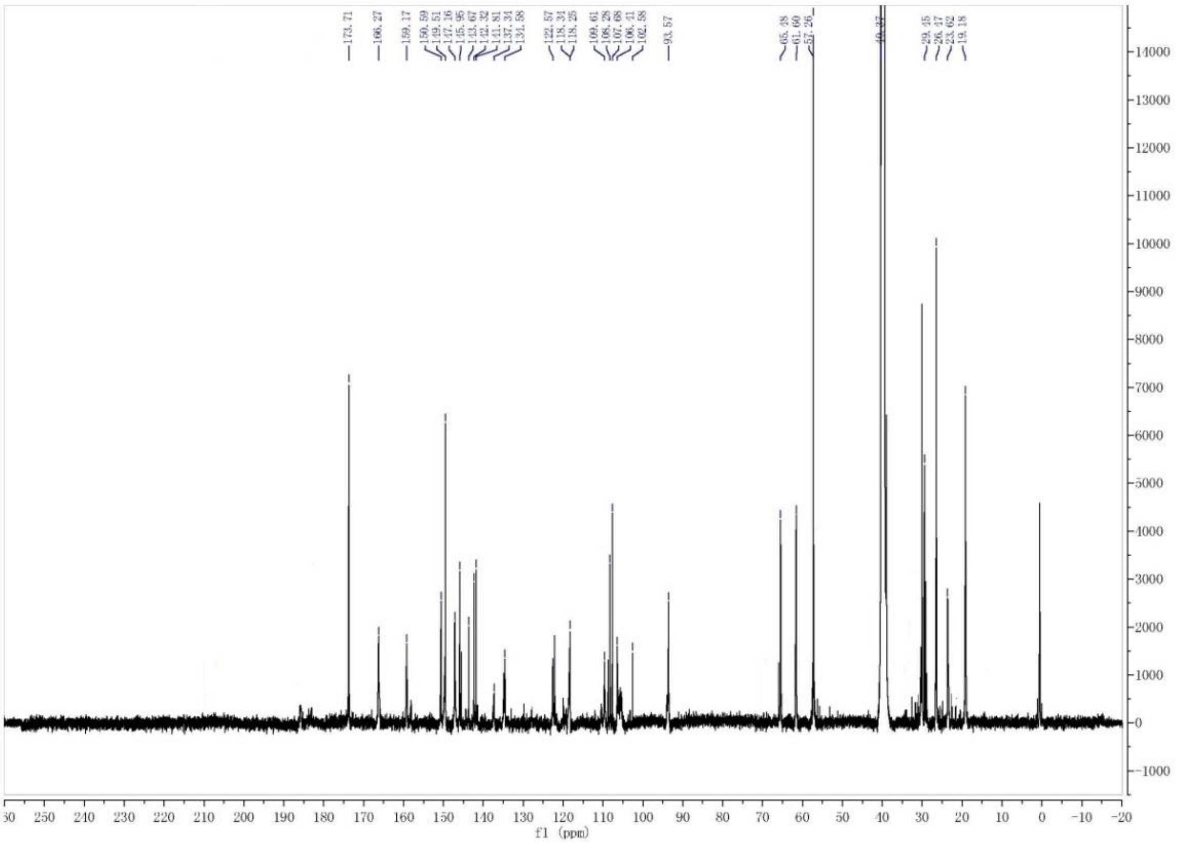

Figure S4. HSQC spectrum of **1** in DMSO-d_6.

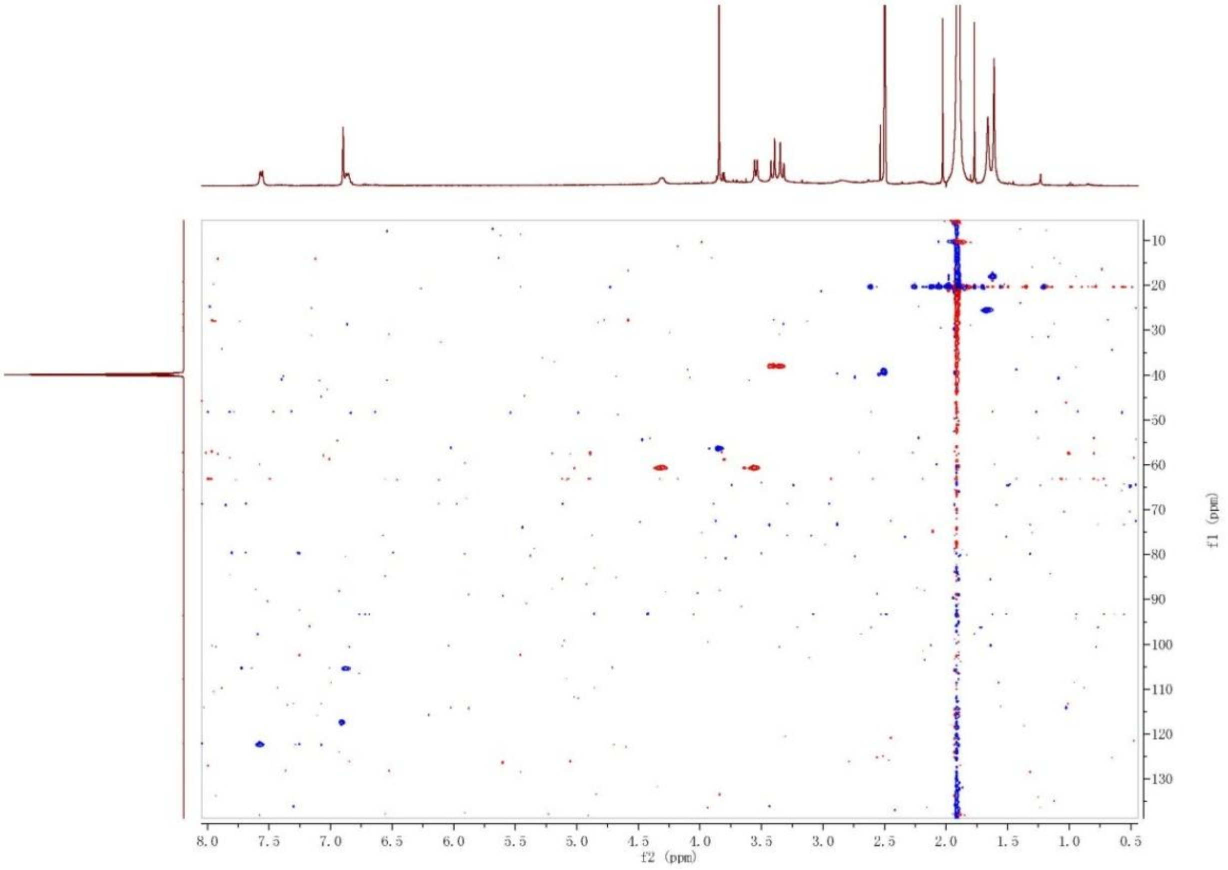

Figure S5. HMBC spectrum of **1** in DMSO-d_6.

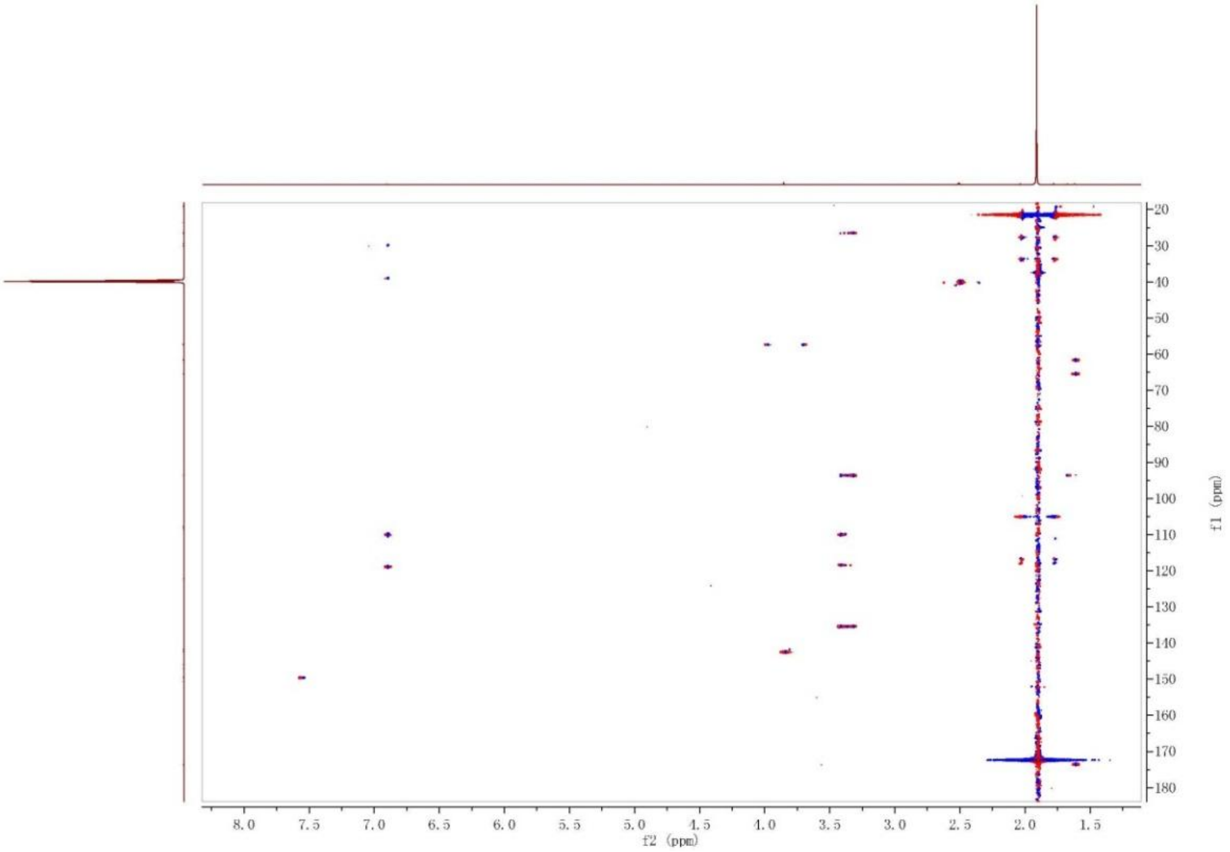

Figure S6. NOESY spectrum of **1** in DMSO-d_6.

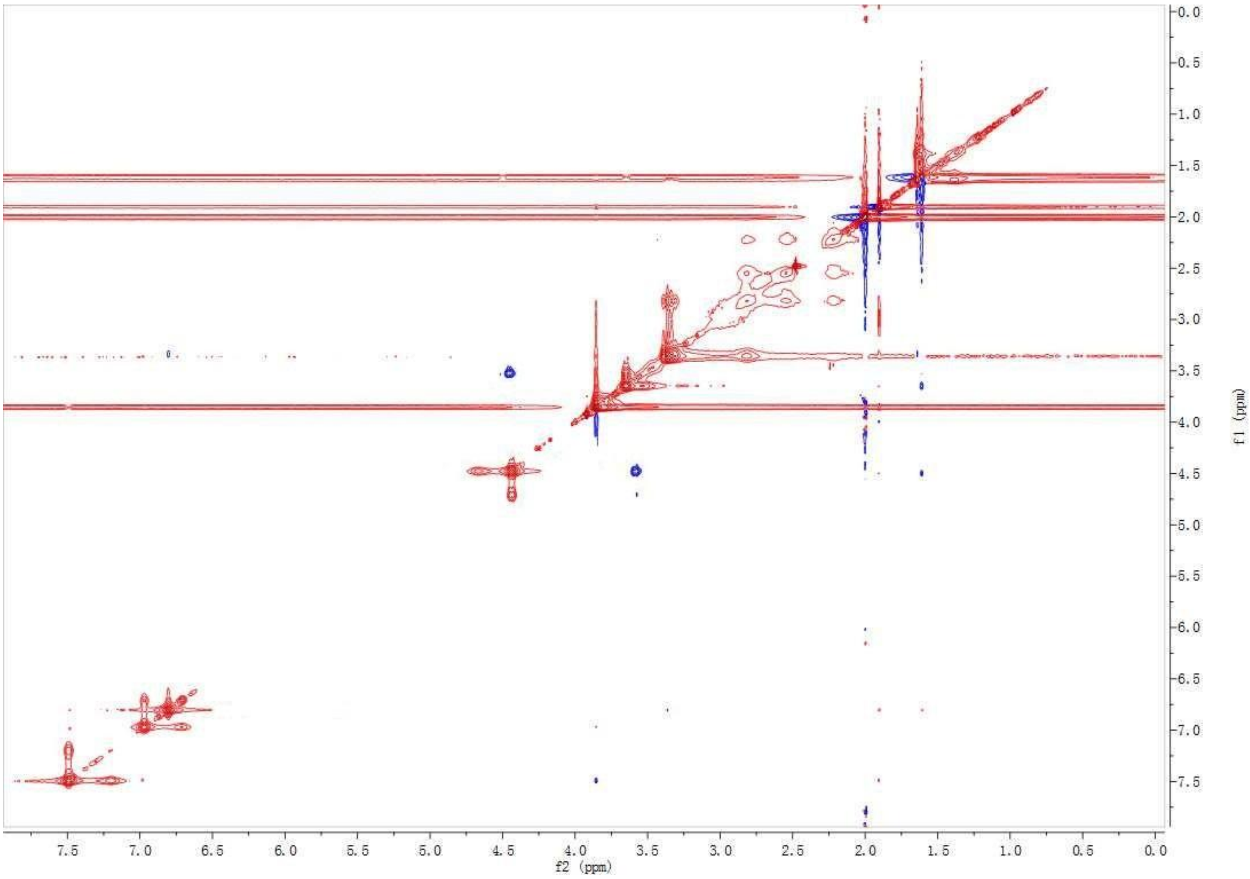

Figure S7. HRESIMS spectrum of **1**.

Display Report

Analysis Info

Analysis Name	G:\leonie-MS\0803\AM-5A_1-C,2_01_3348.d	Acquisition Date	8/3/2011 6:42:48 PM
Method	LC-5%-ms-pos.m	Operator	BDAL@DE
Sample Name	AM-5A	Instrument / Ser#	micrOTOF 10249
Comment			

Acquisition Parameter

Source Type	ESI	Ion Polarity	Positive	Set Nebulizer	4.0 Bar
Focus	Active			Set Dry Heater	200 °C
Scan Begin	50 m/z	Set Capillary	4500 V	Set Dry Gas	8.0 l/min
Scan End	1500 m/z	Set End Plate Offset	-500 V	Set Divert Valve	Waste

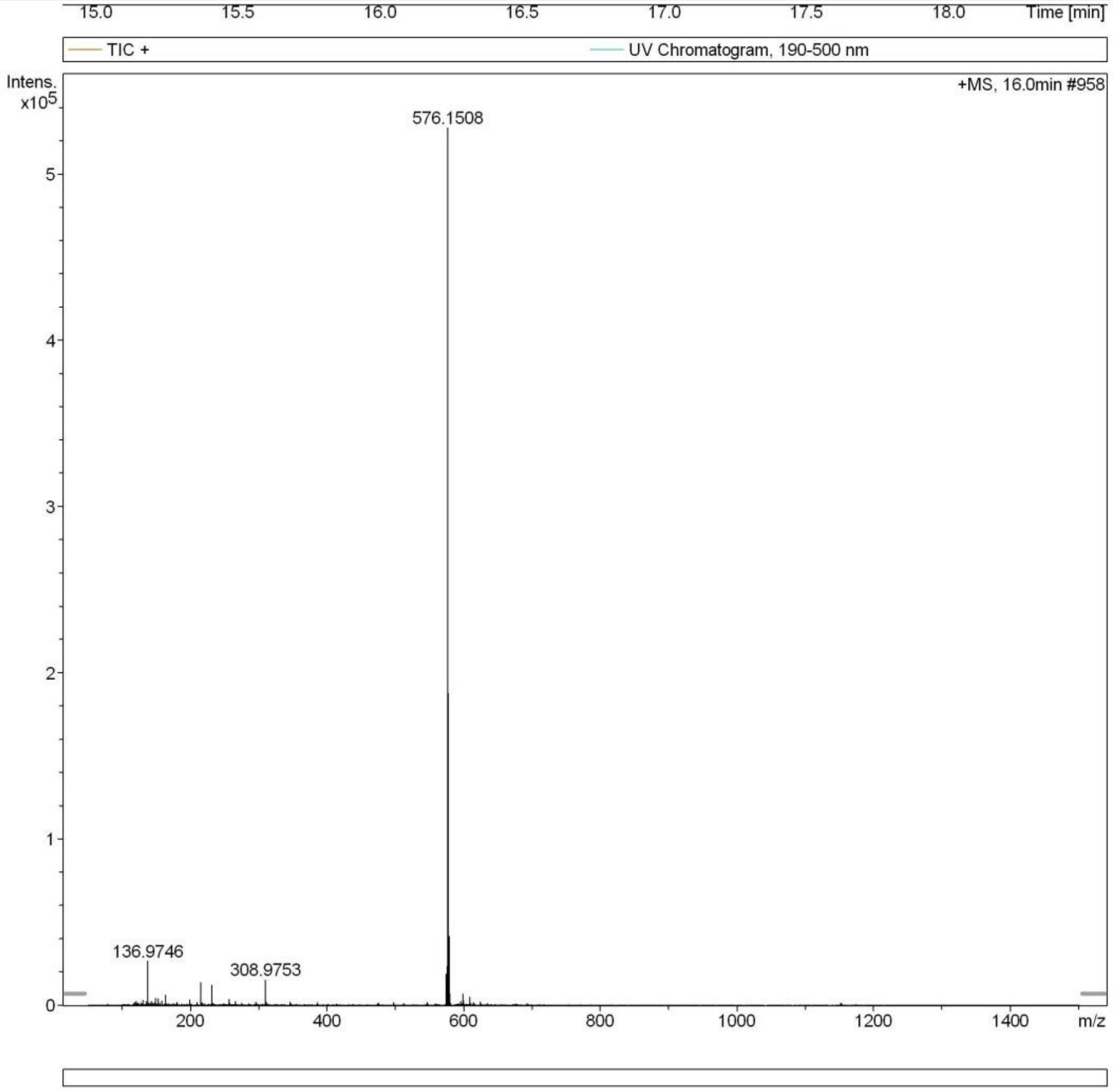

Bruker Compass DataAnalysis 4.0 printed: 8/22/2011 11:17:05 AM Page 1 of 1

Figure S8. ^{1}H NMR spectrum of **2** in DMSO-d_6.

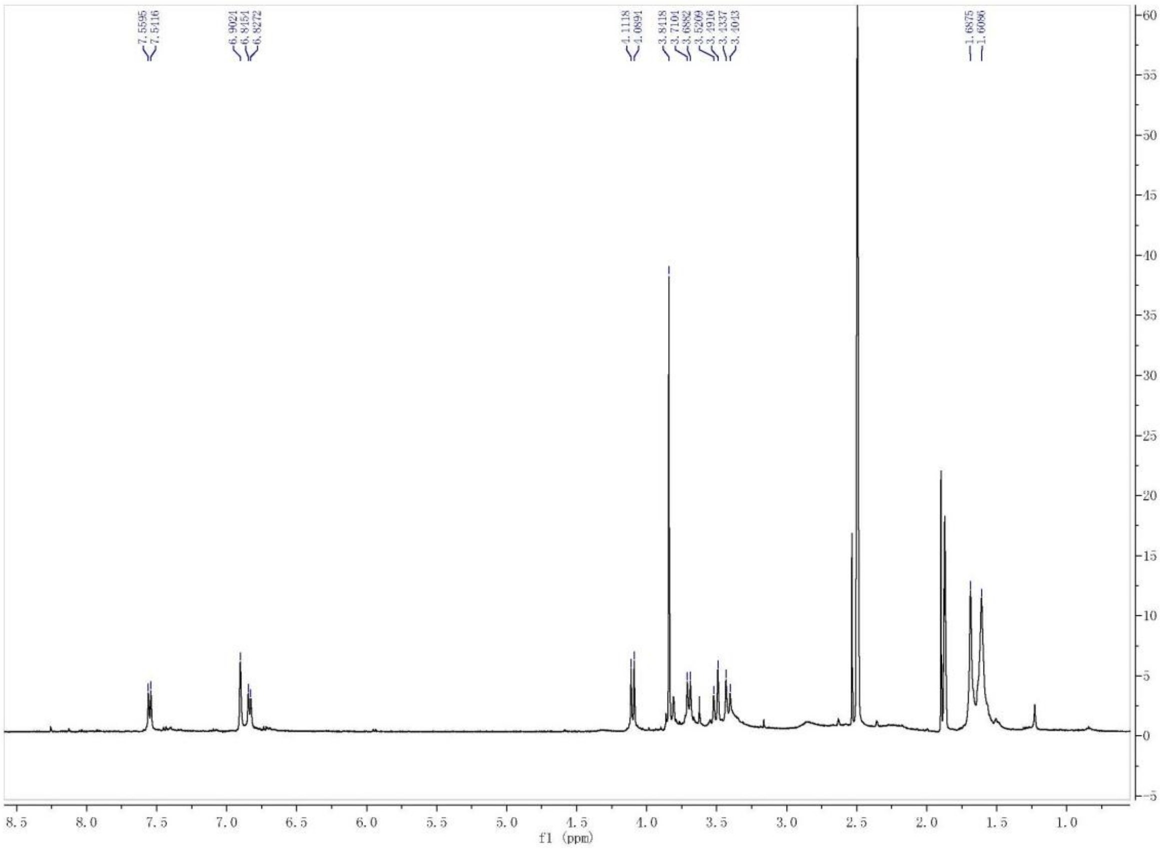

Figure S9. ^{13}C NMR spectra of **2** in DMSO-d_6.

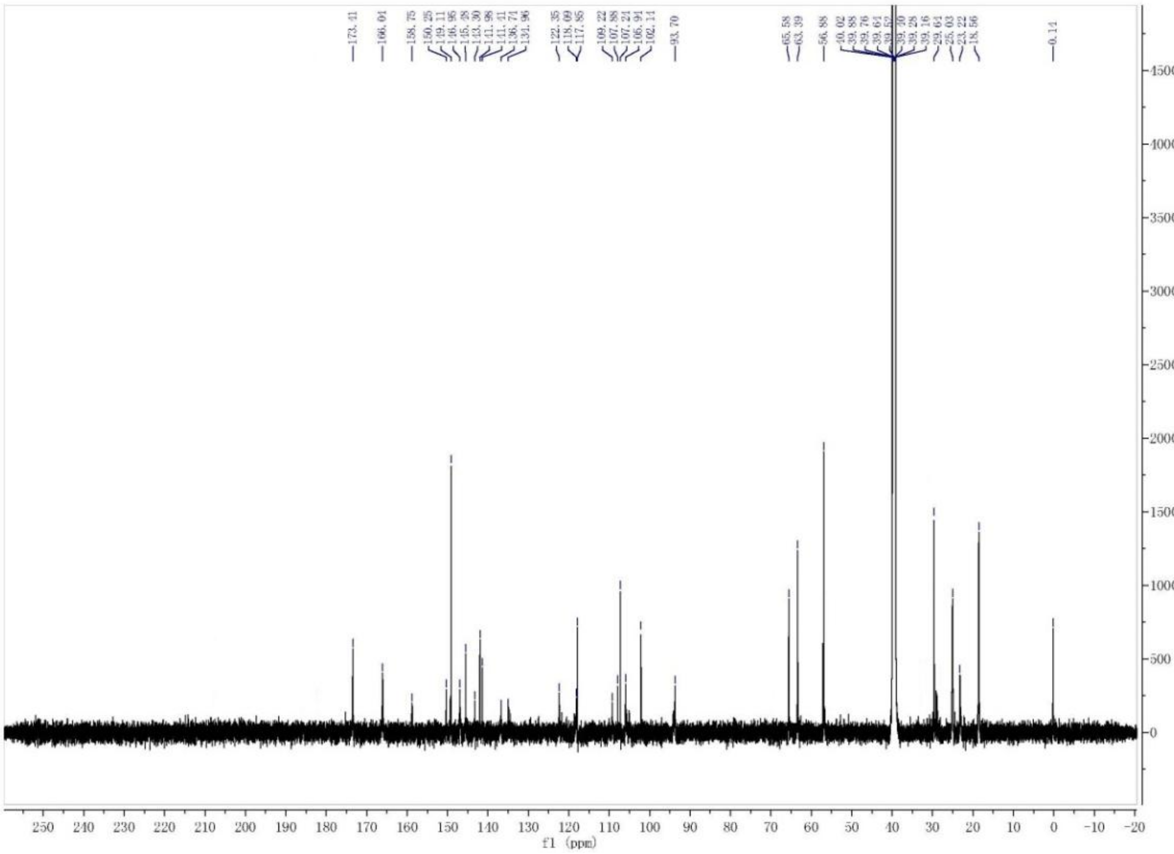

Figure S10. HSQC spectrum of **2** in DMSO-d_6.

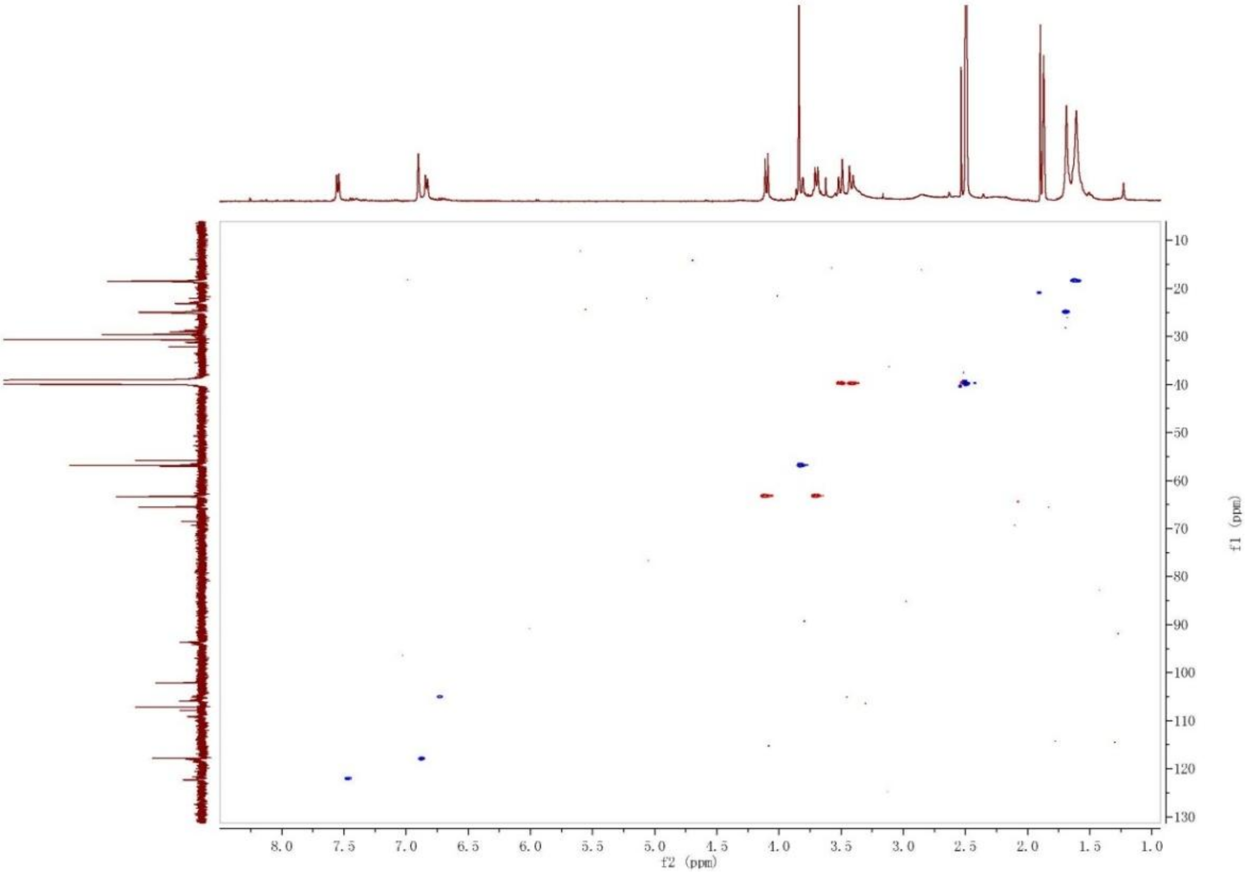

Figure S11. HMBC spectrum of **2** in DMSO-d_6.

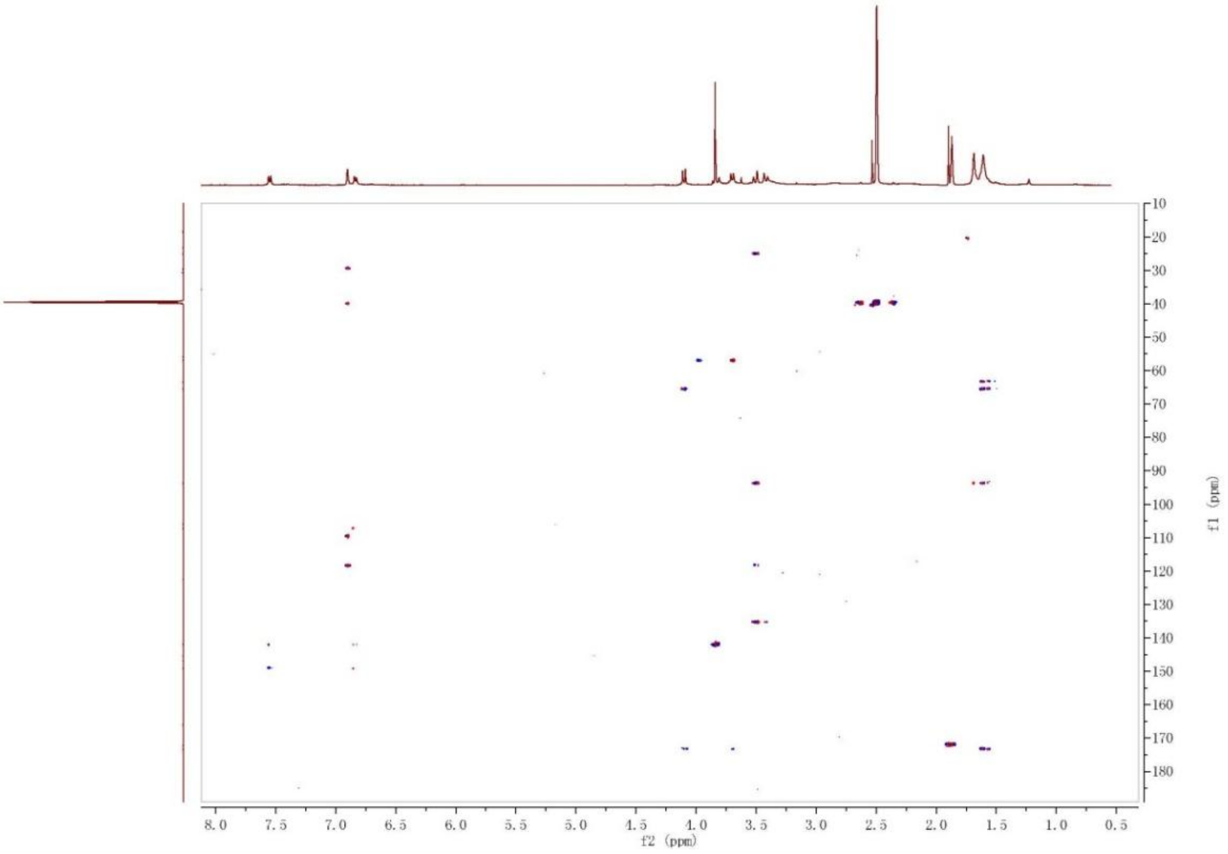

Figure S12. NOESY spectrum of **2** in DMSO-d_6.

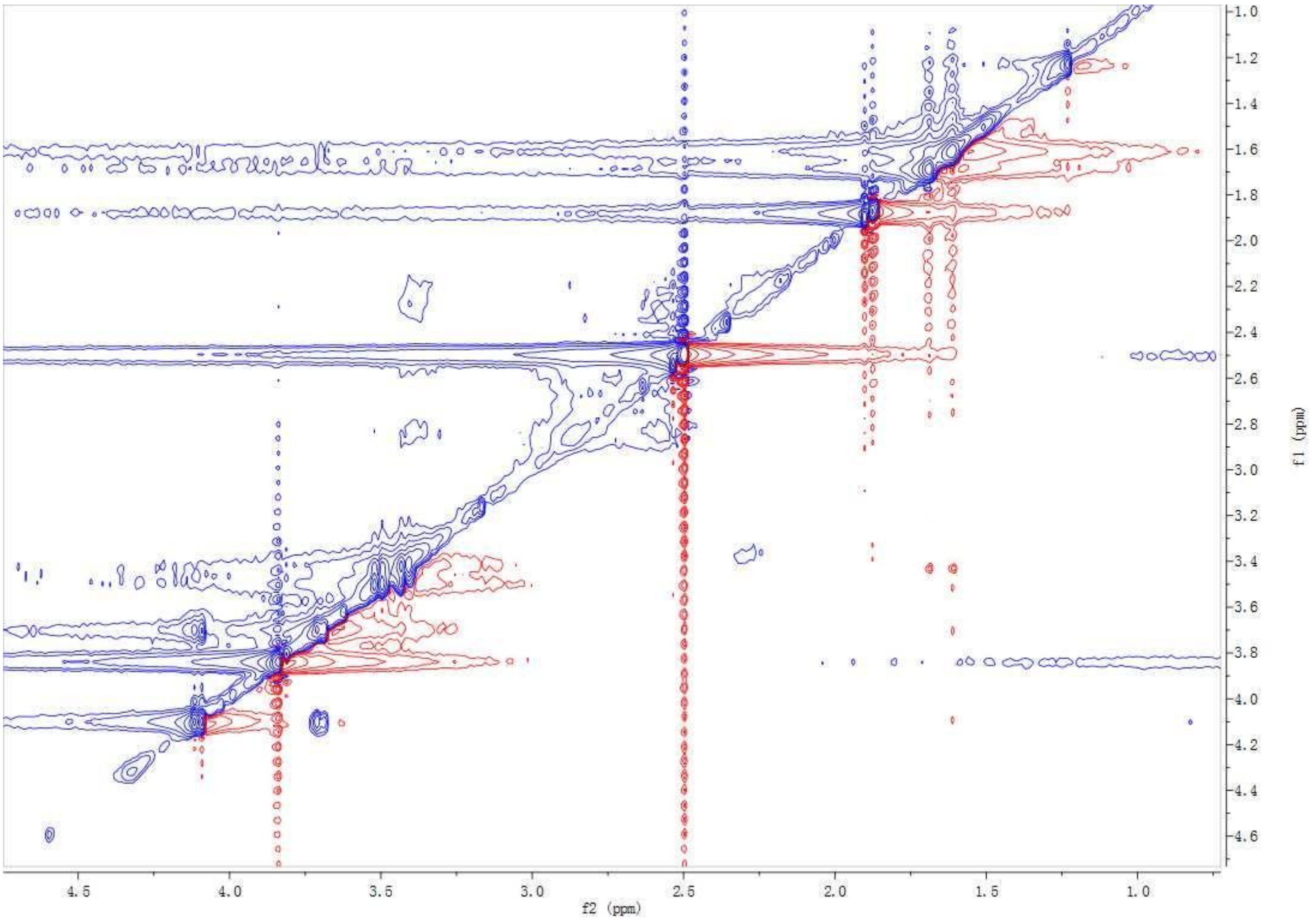

Figure S13. HRESIMS spectrum of **2**.

Display Report

Analysis Info

Analysis Name	G:\leonie-MS\0803\AM-6A_1-C,3_01_3349.d	Acquisition Date	8/3/2011 7:11:06 PM
Method	LC-5%-ms-pos.m	Operator	BDAL@DE
Sample Name	AM-6A	Instrument / Ser#	micrOTOF 10249
Comment			

Acquisition Parameter

Source Type	ESI	Ion Polarity	Positive	Set Nebulizer	4.0 Bar
Focus	Active			Set Dry Heater	200 °C
Scan Begin	50 m/z	Set Capillary	4500 V	Set Dry Gas	8.0 l/min
Scan End	1500 m/z	Set End Plate Offset	-500 V	Set Divert Valve	Waste

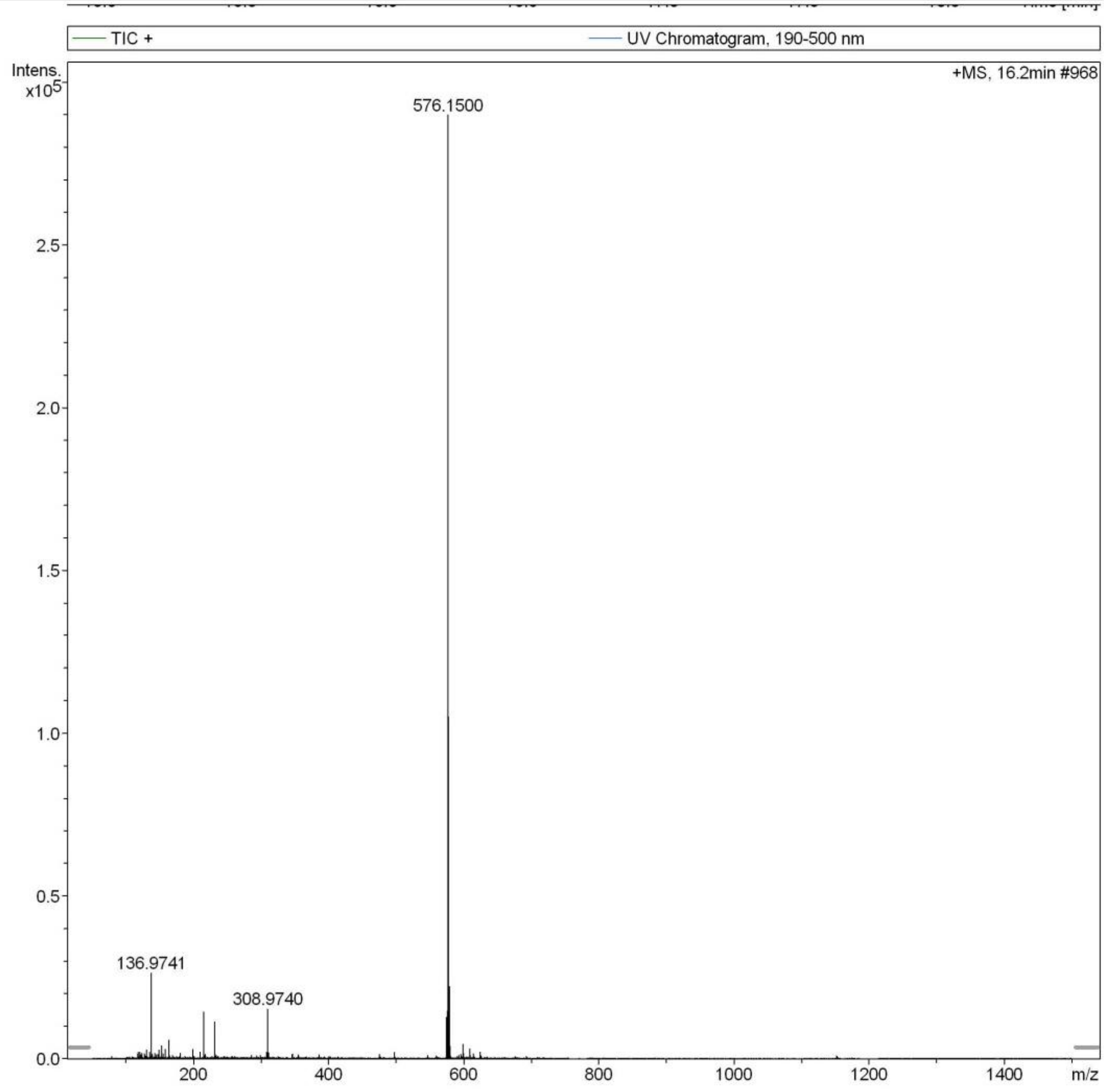

Figure S14. 3D mode and key NOE correlations of compounds **1** and **2**. The structures of compounds **1** and **2** have been optimized by the MM2 force field.

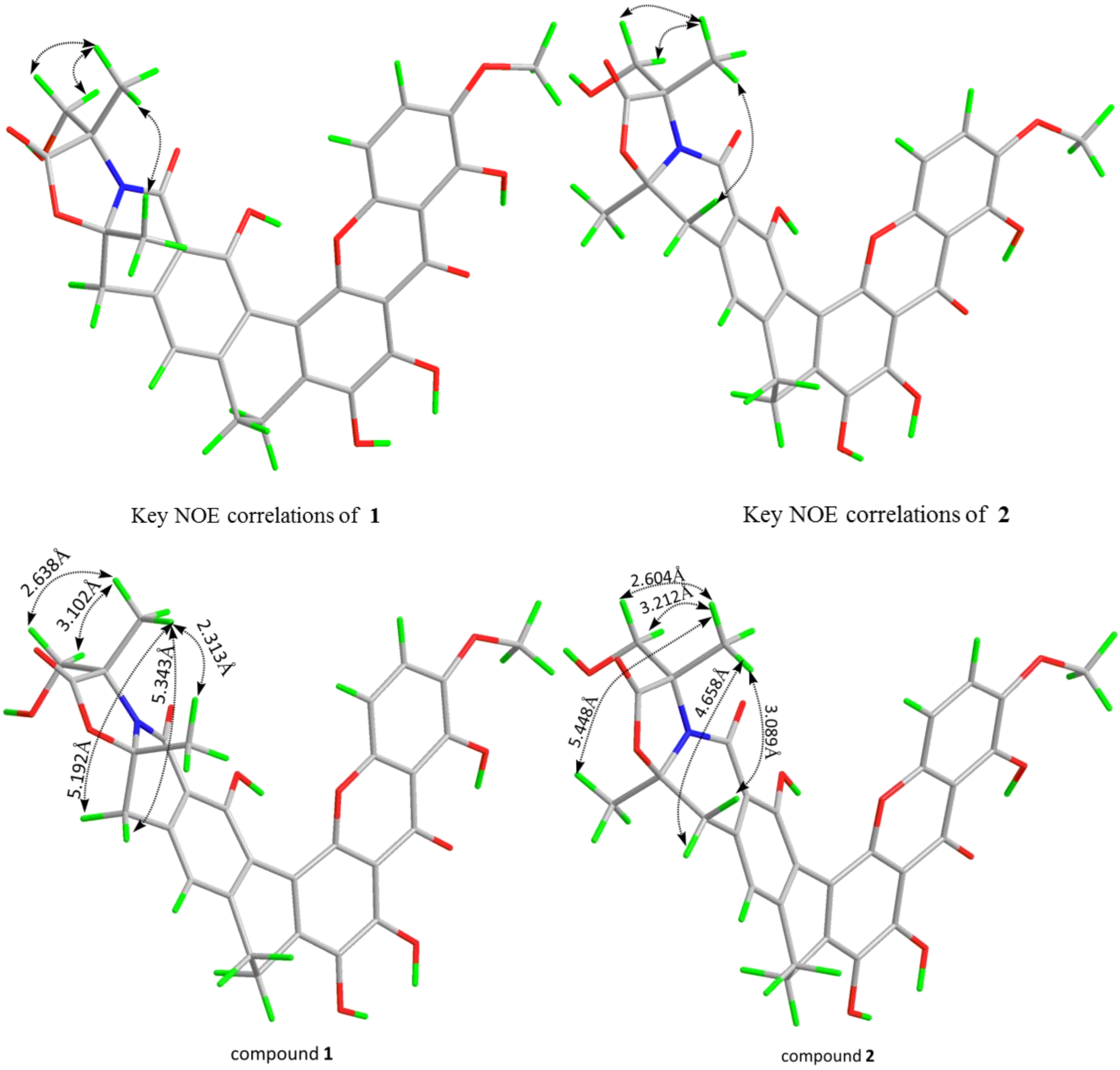

Figure S15. ^{1}H NMR spectrum of **3** in DMSO-d_6.

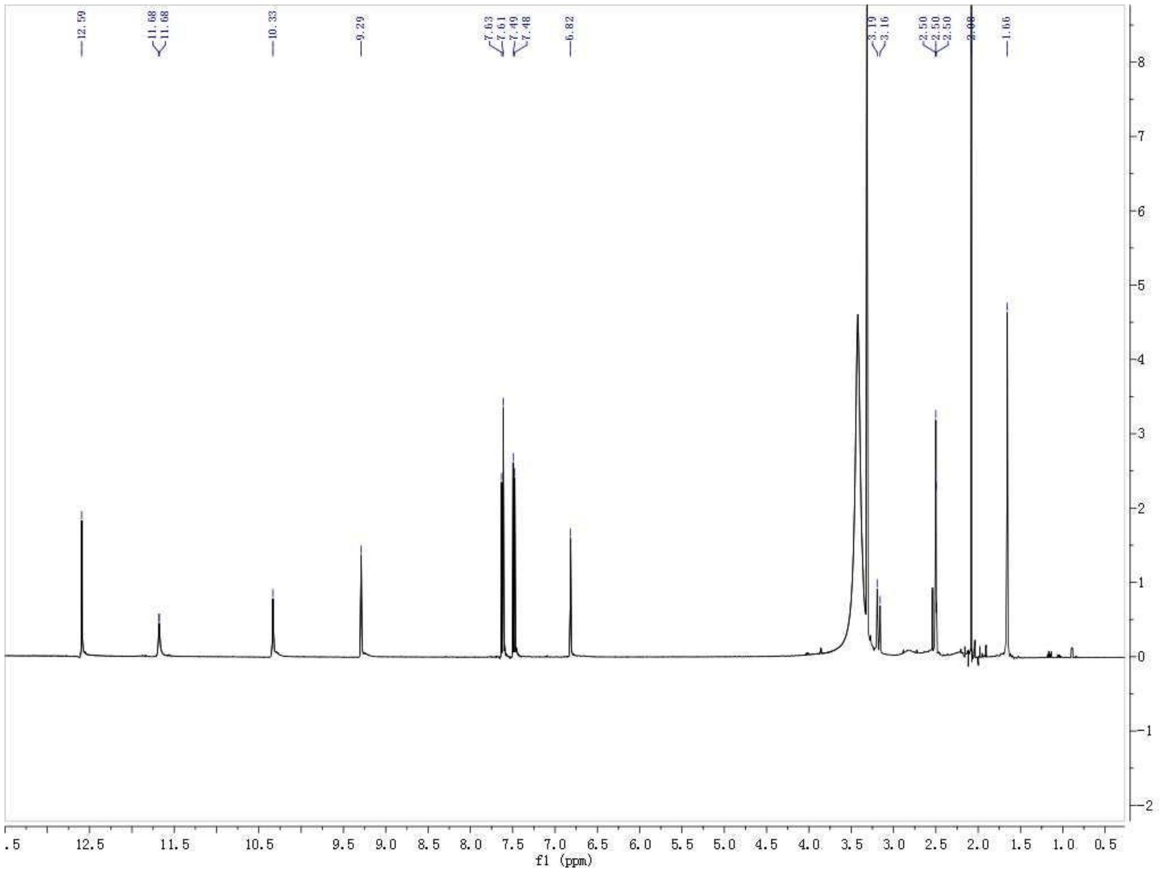

Figure S16. ^{13}C NMR spectrum of **3** in DMSO-d_6.

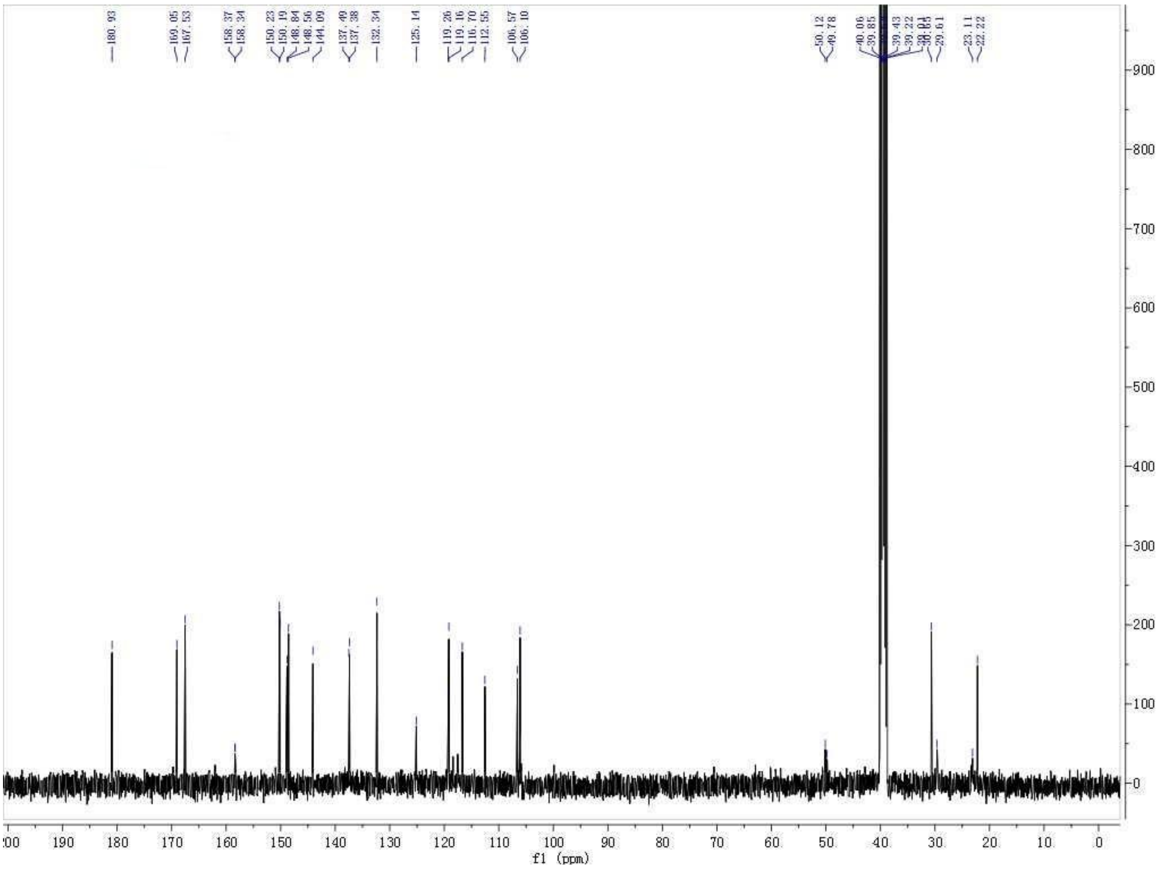

Figure S17. HSQC spectrum of **3** in DMSO-d_6.

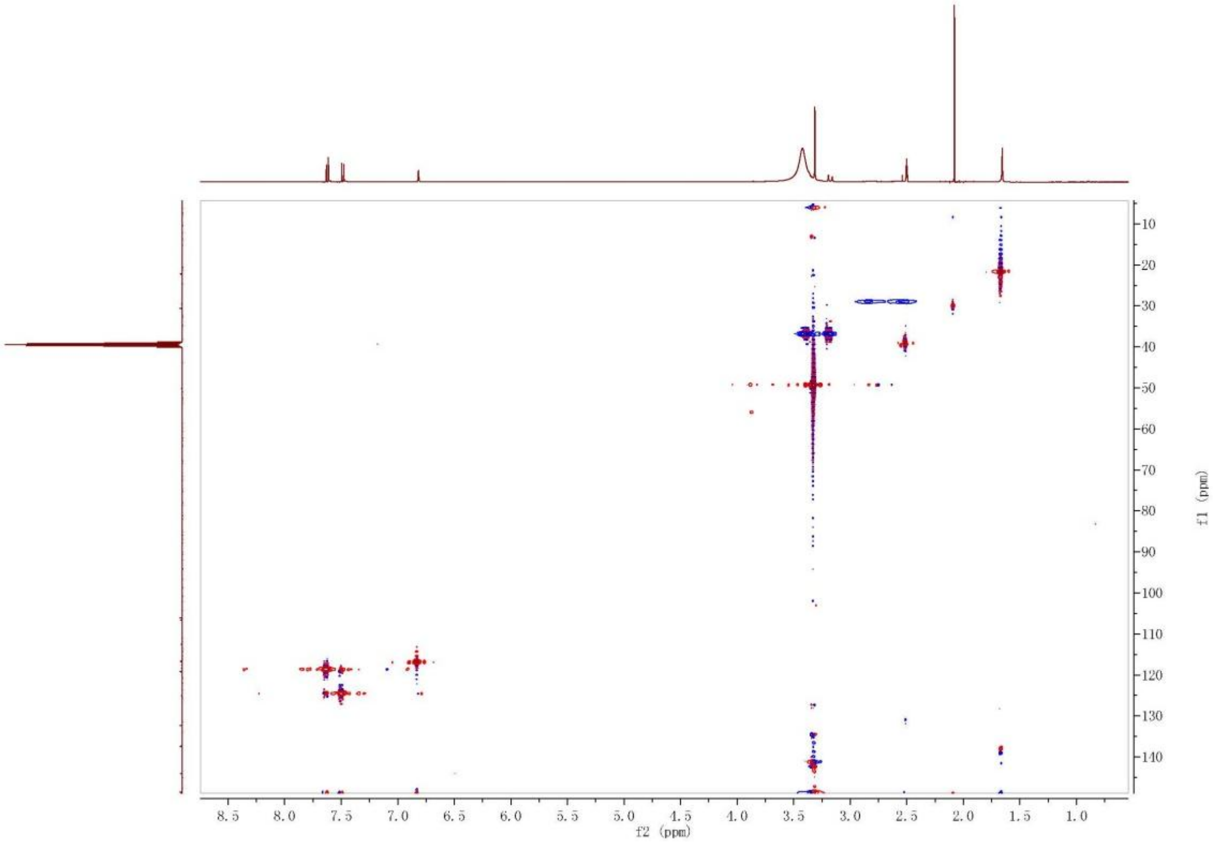

Figure S18. HMBC spectrum of **3** in DMSO-d_6.

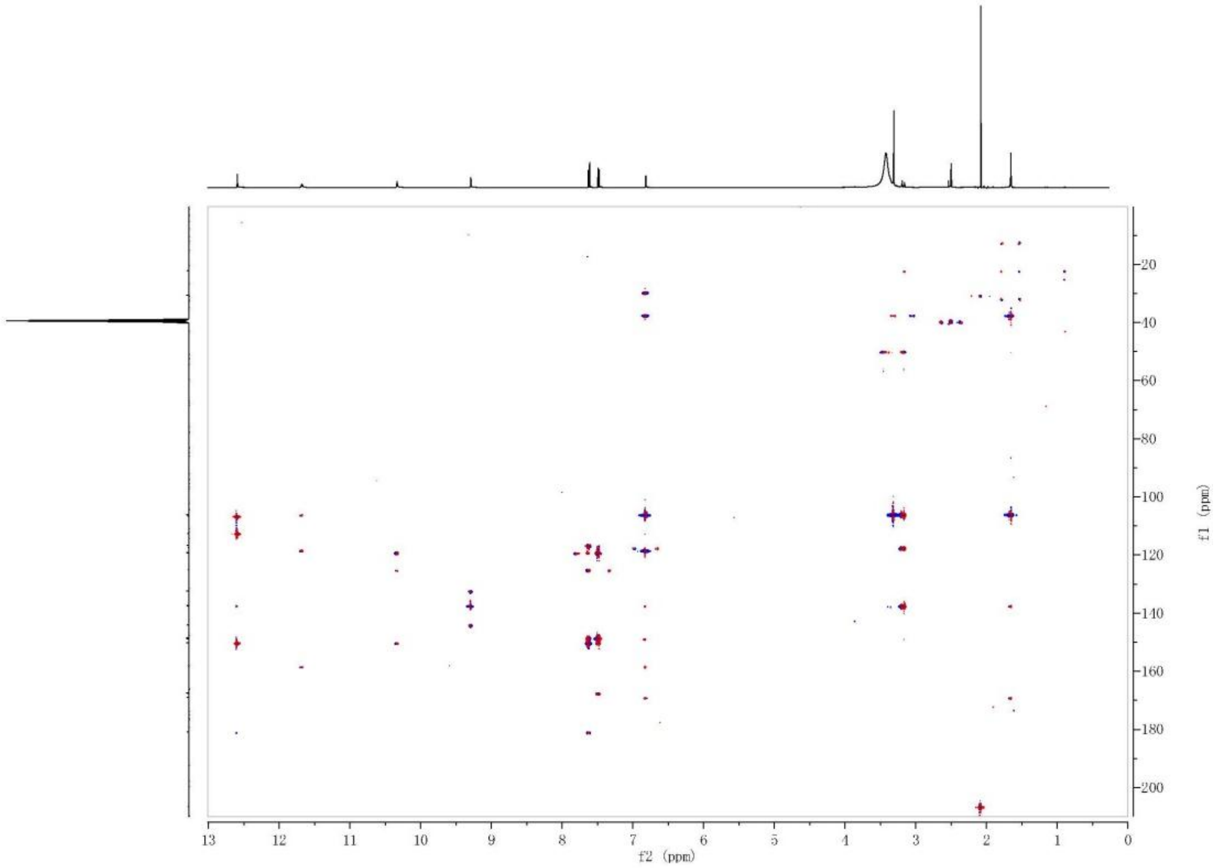

Figure S19. HRESIMS spectrum of **3**.

Display Report

Analysis Info

Analysis Name	G:\leonie-MS\LLL-am-8\AM-8_1-C,1_01_3031.d	Acquisition Date	5/16/2011 11:14:55 PM
Method	LC-MS-5%.m	Operator	BDAL@DE
Sample Name	AM-8	Instrument / Ser#	micrOTOF 10249
Comment			

Acquisition Parameter

Source Type	ESI	Ion Polarity	Positive	Set Nebulizer	4.0 Bar
Focus	Active			Set Dry Heater	200 °C
Scan Begin	50 m/z	Set Capillary	4500 V	Set Dry Gas	8.0 l/min
Scan End	1500 m/z	Set End Plate Offset	-500 V	Set Divert Valve	Waste

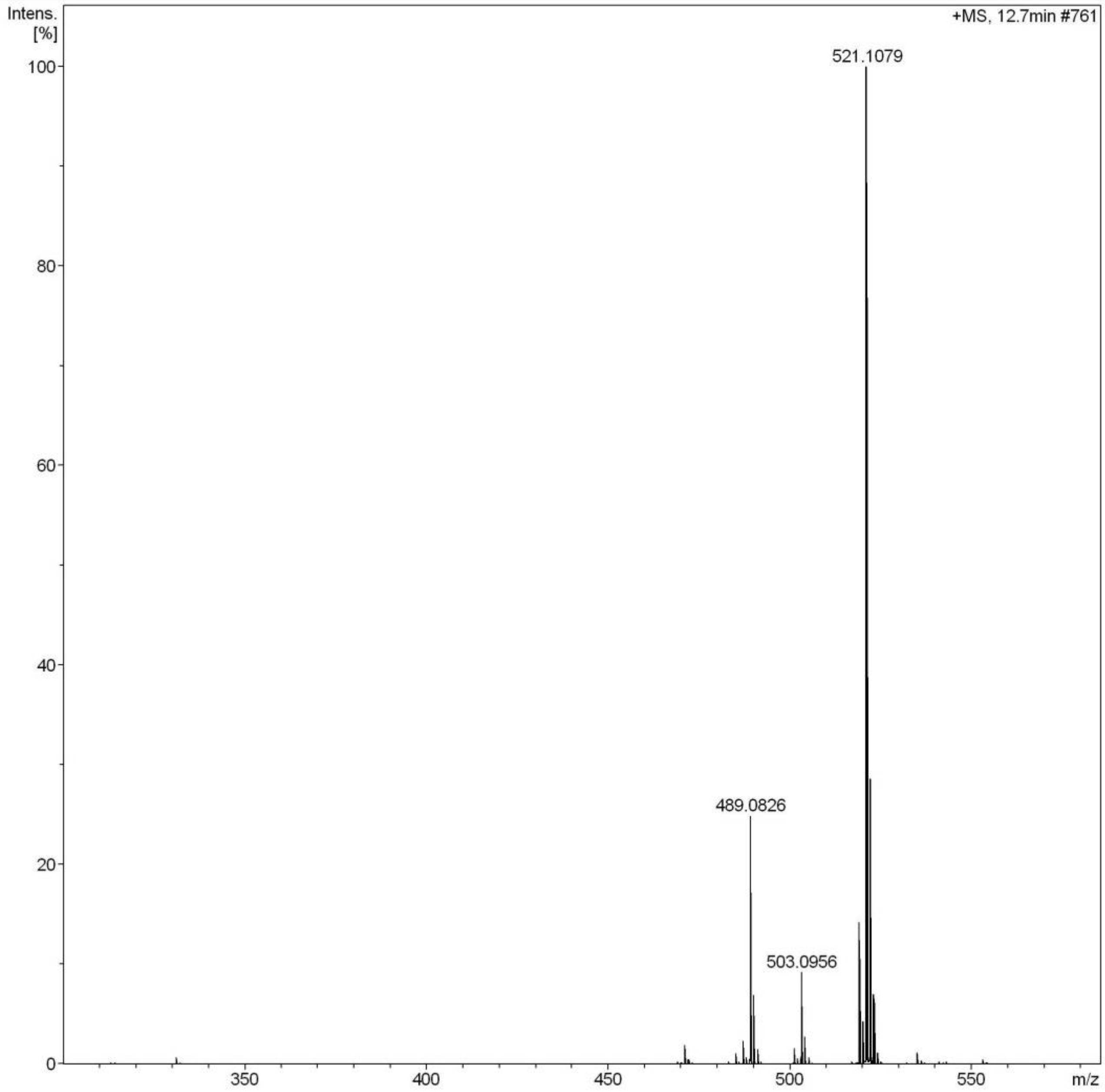

Bruker Compass DataAnalysis 4.0 printed: 1/6/2012 1:37:00 PM Page 1 of 1

Figure S20. ^{1}H NMR spectrum of **4** in DMSO-d_6.

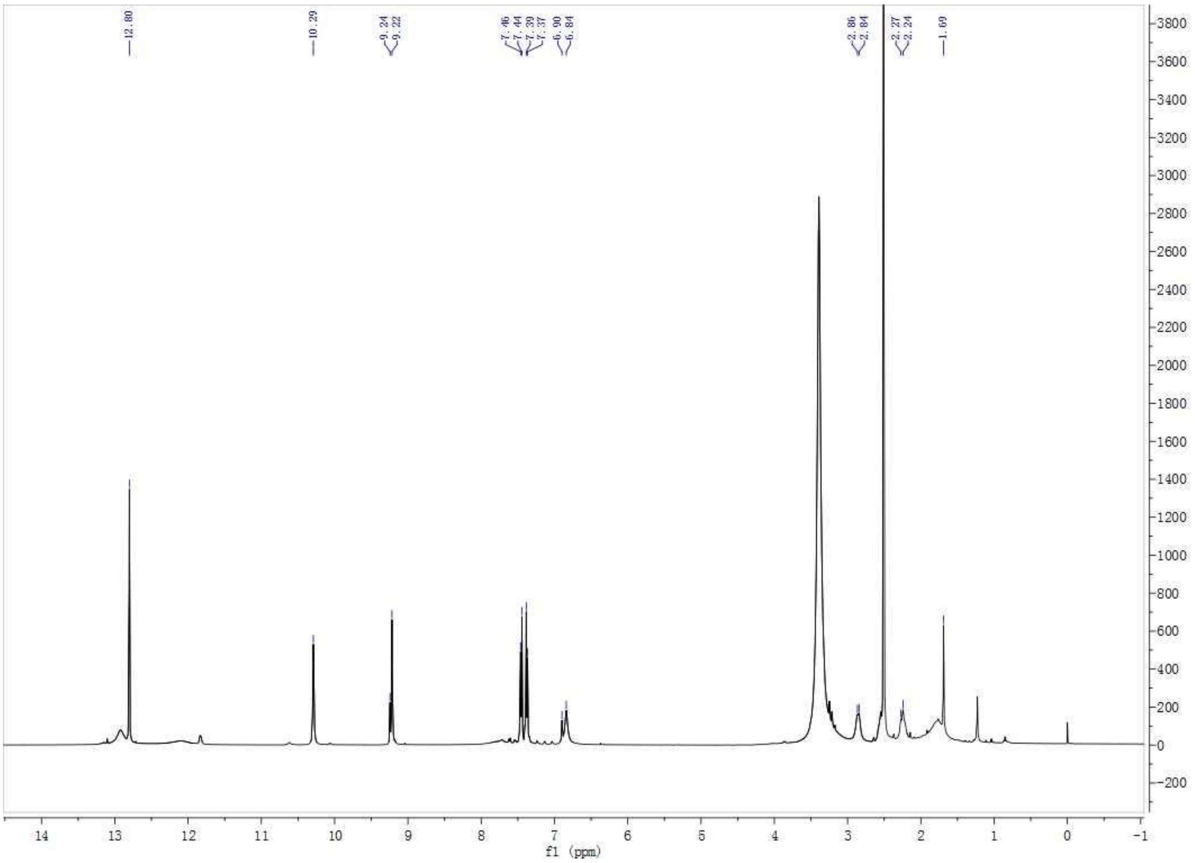

Figure S21. ^{13}C NMR spectrum of **4** in DMSO-d_6.

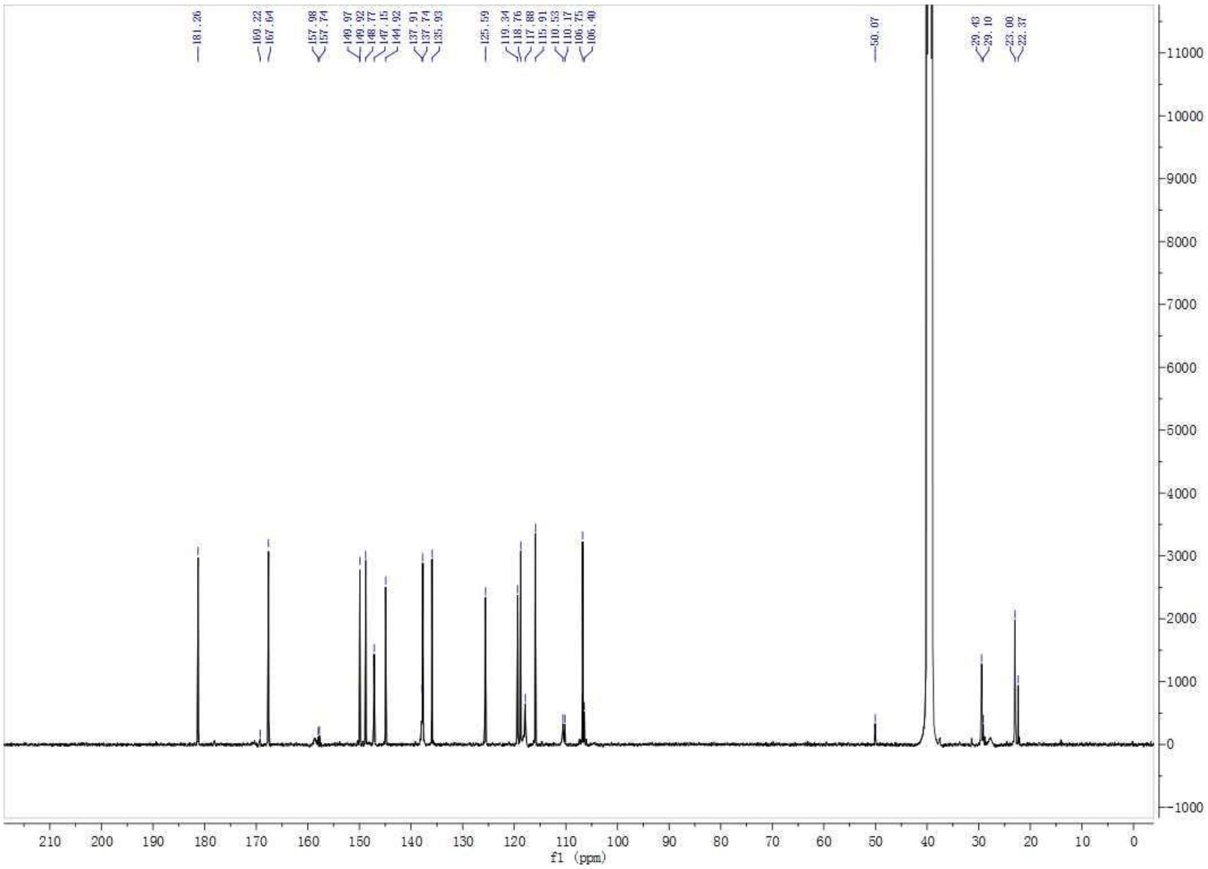

Figure S22. HSQC spectrum of **4** in DMSO-d_6.

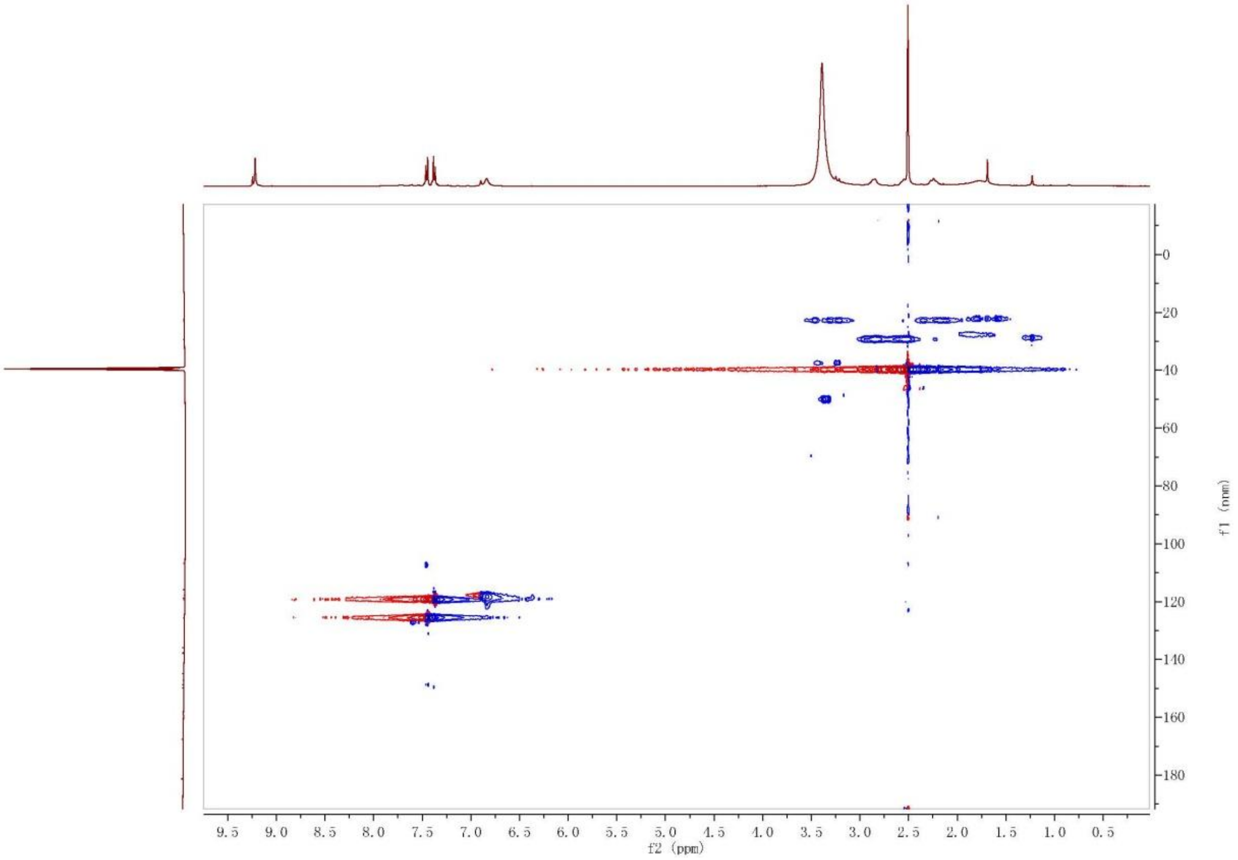

Figure S23. HMBC spectrum of **4** in DMSO-d_6.

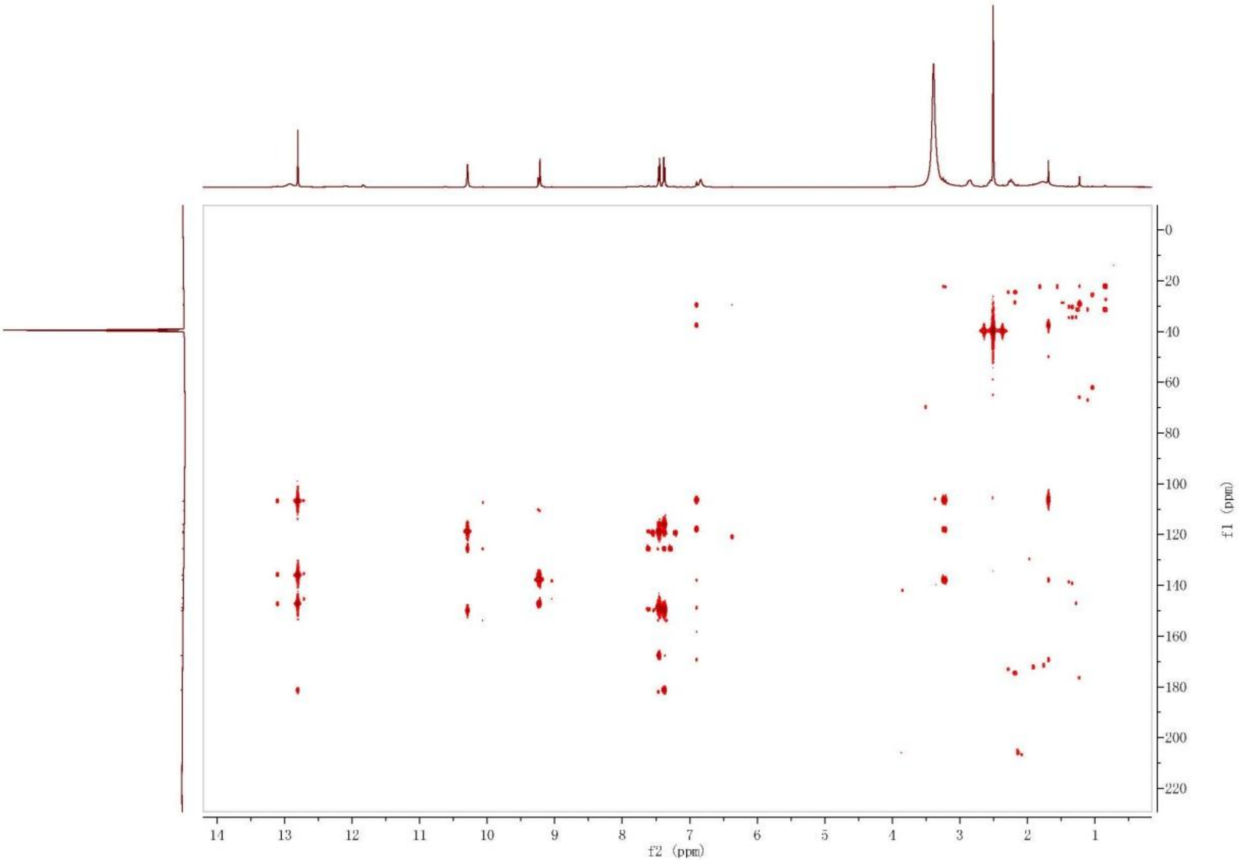

Figure S24. HRESIMS spectrum of **4**.

Display Report

Analysis Info

Analysis Name	G:\leonie-MS\Am-13_1-C,4_01_3059.d	Acquisition Date	5/27/2011 9:26:12 PM
Method	LC-MS-5%.m	Operator	BDAL@DE
Sample Name	Am-13	Instrument / Ser#	micrOTOF 10249
Comment			

Acquisition Parameter

Source Type	ESI	Ion Polarity	Positive	Set Nebulizer	4.0 Bar
Focus	Active			Set Dry Heater	200 °C
Scan Begin	50 m/z	Set Capillary	4500 V	Set Dry Gas	8.0 l/min
Scan End	1500 m/z	Set End Plate Offset	-500 V	Set Divert Valve	Waste

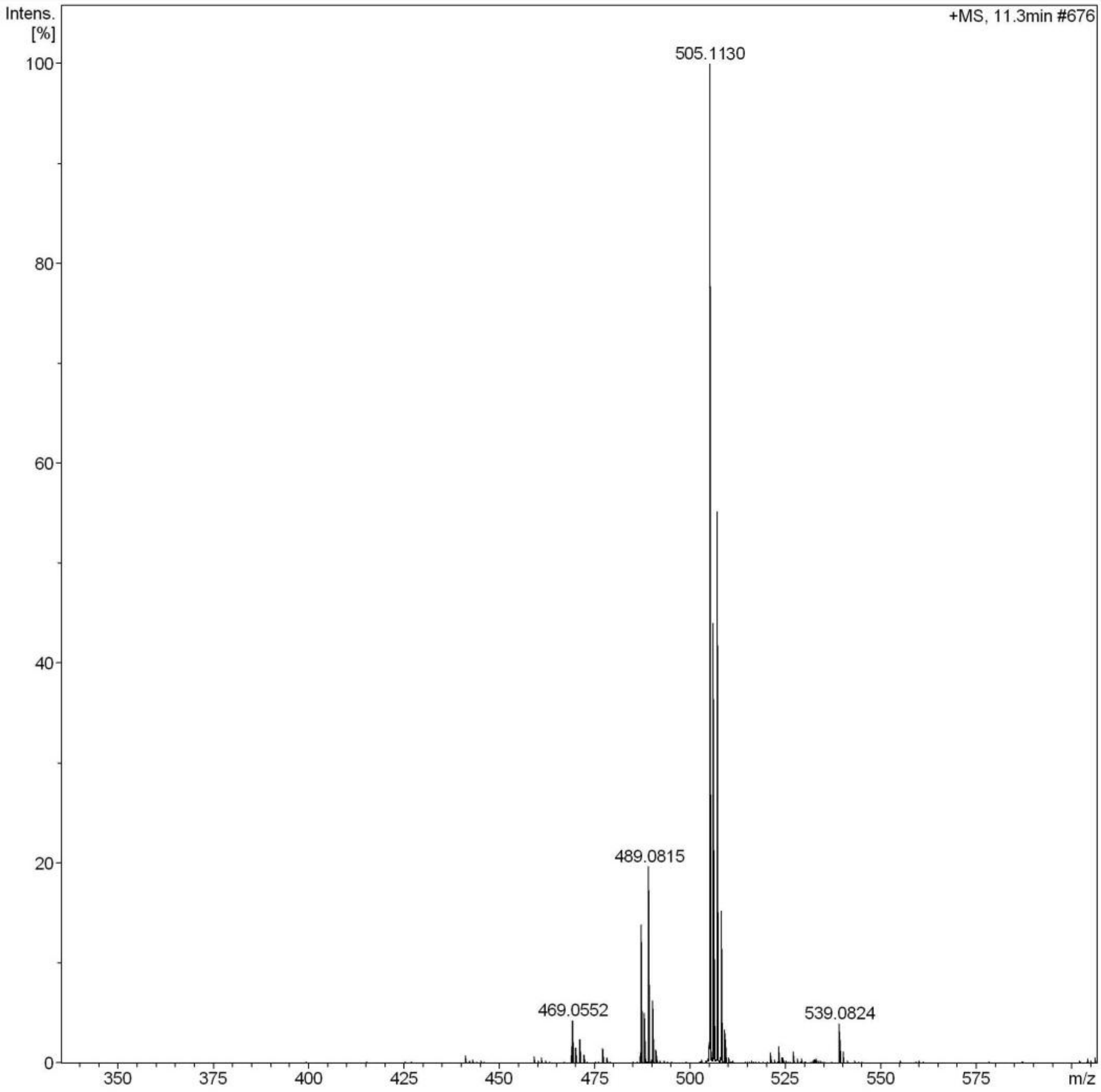

Supplement: Supplementary File 1: — Supplementary Information (PDF, 1628 KB) [file marinedrugs-10-02571-s001.pdf]
